# Fluoroquinolone resistance in complicated urinary tract infections: association with the increased occurrence and diversity of *Escherichia coli* of clonal complex 131, together with ST1193

**DOI:** 10.3389/fcimb.2024.1351618

**Published:** 2024-02-27

**Authors:** Isidro García-Meniño, Vanesa García, Pilar Lumbreras-Iglesias, Javier Fernández, Azucena Mora

**Affiliations:** ^1^ Laboratorio de Referencia de Escherichia coli (LREC), Dpto. de Microbioloxía e Parasitoloxía, Universidade de Santiago de Compostela (USC), Lugo, Spain; ^2^ Instituto de Investigación Sanitaria de Santiago de Compostela (IDIS), Santiago de Compostela, Spain; ^3^ Department for Biological Safety, German Federal Institute for Risk Assessment, Berlin, Germany; ^4^ Servicio de Microbiología, Hospital Universitario Central de Asturias (HUCA), Oviedo, Spain; ^5^ Grupo de Microbiología Traslacional, Instituto de Investigación Sanitaria del Principado de Asturias (ISPA), Oviedo, Spain; ^6^ Research and Innovation, Artificial Intelligence and Statistical Department, Pragmatech AI Solutions, Oviedo, Spain; ^7^ CIBER de Enfermedades Respiratorias (CIBERES), Instituto de Salud Carlos III, Madrid, Spain; ^8^ Departamento de Biología Funcional, Universidad de Oviedo, Oviedo, Spain

**Keywords:** complicated urinary tract infection (cUTI), fluoroquinolone resistance (FQR), uropathogenic *Escherichia coli* (UPEC), ST131, ST1193, ST9126

## Abstract

**Introduction:**

Urinary tract infections (UTIs) are one of the leading causes of multidrug-resistance (MDR) spread and infection-related deaths. *Escherichia coli* is by far the main causative agent. We conducted a prospective study on complicated urinary tract infections (cUTIs) i) to monitor the high-risk clones that could be compromising the therapeutic management and ii) to compare the cUTI etiology with uncomplicated infections (uUTIs) occurring in the same period and health area.

**Methods:**

154 non-duplicated *E. coli* recovered from cUTIs in 2020 at the Hospital Universitario Central de Asturias (Spain) constituted the study collection.

**Results:**

Most cUTI isolates belonged to phylogroup B2 (72.1%) and met the uropathogenic (UPEC) status (69.5%) (≥3 of *chuA*, *fyuA*, *vat*, and *yfcV* genes). MDR was exhibited by 35.7% of the isolates, similarly to data observed in the uUTI collection. A significant difference observed in cUTI was the higher level of fluoroquinolone resistance (FQR) (47.4%), where the pandemic clonal groups B2-CC131 and B2-ST1193 (CH14-64) comprised 28% of the 154 *E. coli*, representing 52.1% of the FQR isolates. Other prevalent FQR clones were D-ST69 (CH35-27), D-ST405 (CH37-27), and B2-ST429 (CH40-20) (three isolates each). We uncovered an increased genetic and genomic diversity of the CC131: 10 different virotypes, 8 clonotypes (CH), and 2 STs. The presence of *bla*_CTX-M-15_ was determined in 12 (7.8%) isolates (all CC131), which showed 10 different core genome (cg)STs and 2 *fim*H types (*fim*H30 and *fim*H602) but the same set of chromosomal mutations conferring FQR (*gyrA* p.S83L, *gyrA* p.D87N, *parC* p.S80I, *parC* p.E84V, and *parE* p.I529L). In addition, the plasmidome analysis revealed 10 different IncF formulae in CC131 genomes.

**Conclusion:**

We proved here that non-lactose fermenting screening, together with the detection of O25b (*rfb*O25b), H4 (*fliC_H4_*), and H5 (*fliC_H5_*) genes, and phylogroup and clonotyping assignation, is a reasonable approach that can be easily implemented for the surveillance of emerging high-risk clones associated with FQR spread in cUTIs, such as the uncommonly reported O25b:H4-B2-ST9126-CC131 (CH1267-30). Since *E. coli* CC131 and ST1193 are also involved in the community uUTIs of this health area, interventions to eradicate these MDR clones, along with surveillance for other emerging ones, are essential for antibiotic use optimization programs.

## 1 Introduction

Urinary tract infection (UTI) is a leading cause of community-acquired and nosocomial infections, accounting for 30%–40% of all infections treated in hospitals (Klevens et al., 2007), and a second cause of antibiotic prescription globally. In addition to their important public health burden, affecting in the USA more than 150 million individuals annually, with more than 10 million outpatient visits per year and approximately 100,000 hospital admissions, UTIs also pose a high economic impact with an estimated cost between 1.6 and 3.5 billion dollars every year (Flores-Mireles et al., 2015; MacVane et al., 2015; Aabenhus et al., 2017; Medina and Castillo-Pino, 2019; Klein and Hultgren, 2020).

The global number of deaths associated with UTI across 11 infectious syndromes and 33 bacterial pathogens is also outstanding, which accounted for an estimation of 375,106 deaths in 2019, with 14.1 deaths per 100,000 habitants only in Spain (Ikuta et al., 2022). In women, the anatomy and shorter urethra facilitate the increased susceptibility to UTIs, thus being more susceptible to suffer UTI than men. In fact, over 50% of women will develop at least one episode of UTI at some point in their lifetime, and one in three will have at least one symptomatic UTI requiring antimicrobial treatment by the age of 24 (Foxman, 2003; Aydin et al., 2015).

The clinical spectrum of UTIs comprises a heterogeneous group of conditions, differing from uncomplicated UTI (uUTI) to complicated UTI (cUTI). The uUTI is relatively frequent in healthy, premenopausal, sexually active women. On the contrary, the cUTI occurs in patients with structural abnormalities or underlying diseases, which increases the chance to progress to a severe infection (acute and/or chronic renal failure affecting the function of kidneys, and even urosepsis), requiring longer antibiotic treatments (Foxman, 2010; Gupta et al., 2011). Finally, the recurrent UTI (rUTI) also represents an important clinical problem, where pathogen persistence in the gut microbiota, or in the bladder epithelium, causes at least nearly half of the patients getting a second infection over the course of a year (Aydin et al., 2015).

Uropathogenic *E. coli* (UPEC) is by far the main etiological agent diagnosed in all kinds of UTIs (75%–95%) (Foxman, 2014; Flores-Mireles et al., 2015; Medina and Castillo-Pino, 2019), and their health impact was estimated in approximately 120,000 deaths globally during 2019 (UTIs and pyelonephritis by UPEC), which mostly were associated with antimicrobial resistance (AMR) (Ikuta et al., 2022; Murray et al., 2022). Successful multidrug-resistant (MDR) *E. coli* lineages, also known as high-risk clones, such as ST38, ST131, ST167, ST405, ST410, ST648, or ST1193, are frequently reported in these infections (Manges et al., 2019). Together with AMR genes, they are typically carriers of a broad arsenal of extraintestinal virulence factors, which contributes to their pathogenicity. Among them, ST131 is recognized as the most successful, and ST1193 seems to follow in the footsteps of ST131 (Pitout et al., 2022). These disseminated MDR clones seem to be associated with fluoroquinolone resistance (FQR), and many are also producers of CTX-M enzymes, which seriously narrows the treatment options (Mamani et al., 2019; Flament-Simon et al., 2020a; Cummins et al., 2021; Pitout et al., 2022). As stated by other authors, evolutionary, surveillance, and clinical studies are urgently required to investigate the success of emerging clones such as ST1193 for management and prevention strategies (Pitout et al., 2022).

In a previous study, we uncovered the presence of FQR ST1193 clone implicated in 6% of the uUTI, which represents the first report in Spain in this pathology. The genomic analysis showed similar key traits than those ST1193 clones disseminated worldwide (García-Meniño et al., 2022). We aimed here i) to identify high-risk *E. coli* clones implicated in cUTI that could be compromising therapeutic management and ii) to compare the cUTI clones with uUTI occurring in the same health area (Oviedo, Spain) and sampling period (2020) as the cUTI collection of the present study.

## 2 Methods

### 2.1 cUTI *E. coli* collection

We conducted a prospective specific study on cUTI, which included 154 non-duplicated *E. coli* isolates recovered from urine samples collected between January 2020 and March 2020. The distribution of the samples by gender was as follows: 108 women with an age range between 8 months and 97 years old, and 46 men between 3 months and 95 years old (median =71; interquartile range, IQR = 30) (**Supplementary Table S1**). Uncomplicated infections, defined as those occurring in young women without any functional or anatomical anomalies in the urinary tract, were excluded from the study. The primo isolation from urine samples was performed in the Hospital Universitario Central de Asturias (HUCA) in northern Spain, whose health area covers a population of approximately 300,000 persons.

Bacterial identification was performed by matrix-assisted laser desorption/ionization–time-of-flight mass spectrometry (MALDI-TOF) (Bruker Daltonik, Bremen, Germany) after conventional culture in CHROMID™ CPS^®^ Elite (BioMérieux,Marcy L’Étoile, France). A reliable result (at the species level) was only considered if the score obtained was higher than 2. The recovered isolates were stored at room temperature in nutrient broth (Difco) with 0.75% nutrient agar (Difco) for further characterization in the Reference Laboratory for *E. coli* (LREC-USC).

### 2.2 Lactose fermenting assessment

At the LREC-USC, the collection was grown by streaking on MacConkey Lactose agar (ML) (Oxoid), 37°C overnight. Non-lactose fermenting (NLF) *E. coli* were phenotypically identified by their inability to ferment lactose on the ML agar.

### 2.3 PCR screening of virulence traits

One single colony from the previous step was plated onto Tryptone Soy Agar (Oxoid) at 37°C overnight. Then, the bacterial growth was picked with a 1-µl inoculation loop and suspended in 600 µl of sterile Milli-Q water. Bacterial suspensions were boiled at 100°C for 5 min and then centrifuged for 2 min at 11,000 rpm to pellet bacterial debris. The supernatant was used as DNA template for the polymerase chain reaction (PCR) screening of specific virulence-encoding genes, statistically associated with higher efficiency in the colonization of the urinary tract. According to the results, the so-called status UPEC was assigned to isolates positive for ≥3 of the following marker genes (*chuA*, *fyuA*, *vat*, and *yfcV*) (Spurbeck et al., 2012) (**Supplementary Table S2**). The PCR screening of *rfb*O25 and *fli*C_H4_, was performed to presumptively determine the pandemic ST131 clonal group. Additionally, the flagellar-encoding gene *fli*C_H5_ typically associated with NLF *E. coli* ST1193 was screened in those lactose negative isolates (**Supplementary Table S3**).

### 2.4 Antimicrobial susceptibility testing

Minimal inhibitory concentrations (MICs) of the 154 *E. coli* isolates were obtained using the MicroScan WalkAway System (Beckman Coulter, Brea, CA, USA) against 28 drug/drug combination: penicillins (ampicillin/amoxicillin, piperacillin, and ticarcillin); antipseudomonal penicillins + beta-lactamase inhibitors (piperacillin-tazobactam); penicillins + beta-lactamase inhibitors (amoxicillin-clavulanic acid); narrow spectrum cephalosporins (cefuroxime); broad-spectrum cephalosporins (cefepime, cefixime, cefotaxime, and ceftazidime); broad-spectrum cephalosporins + beta-lactamase inhibitors (ceftazidime-avibactam and ceftolozane-tazobactam); carbapenems (ertapenem, imipenem, and meropenem); monobactams (aztreonam); fluoroquinolones (norfloxacin, ciprofloxacin, and levofloxacin); aminoglycosides (amikacin, gentamicin, and tobramycin); glycylcyclines (tigecycline); nitrofurans (nitrofurantoin); phosphonic acids (fosfomycin); folate pathway inhibitors (trimethoprim, trimethoprim/sulfamethoxazole); and polymyxins (colistin). Additionally, colistin susceptibility was analyzed with the BMD method as described elsewhere (García-Meniño et al., 2020). Susceptibility results were interpreted according to European Committee on Antimicrobial Susceptibility Testing guidelines (EUCAST 2022). Isolates were classified as MDR if they displayed resistance to a drug of three or more of the above-mentioned antimicrobial categories (Magiorakos et al., 2012).

### 2.5 Screening of *mcr* and *bla* genes

The presence of *mcr* and *bla* genes was investigated by PCR, in those isolates phenotypically suspected of being colistin resistant, or extended-spectrum betalactamase (ESBL) producers, respectively (García-Meniño et al., 2021), as described elsewhere (**Supplementary Table S4**).

### 2.6 Phylogroup, clonotype, sequence type, and virotype assignment

The clonal structure of the whole collection was established by means of phylogrouping. Clonotyping was performed for those isolates that were positive for any of the following traits: FQR, O25b:H4, H5, and non-lactose fermenting (Wirth et al., 2006; Weissman et al., 2012; Clermont et al., 2013, 2019). Briefly, the phylogroup was established according to the PCR-based method developed by Clermont et al. (2013, 2019), which allows the rapid identification of the eight *E. coli* phylogroups belonging to *E. coli sensu stricto* (A, B1, B2, C, D, E, F, and G) (**Supplementary Table S5**). The clonotyping scheme utilizes the 489-nucleotide (nt) internal fragment of *fimH* (encoding the type 1 fimbrial adhesin) and the 469-nt internal *fumC* fragment retrieved from standard MLST (Weissman et al., 2012). Then, isolates of the prevalent clonotypes (CH) (determined in ≥ 3 isolates) were fully characterized by MLST following the Achtman scheme of seven genes (*adk*, *fumC*, *gyrB*, *icd*, *mdh*, *purA*, and *recA*) (Wirth et al., 2006) (**Supplementary Table S6**). Finally, CC131 *E. coli* were further typified by means of the virotyping scheme developed by Dahbi et al. (2014) based on the presence or absence of specific extraintestinal virulence factors, which differentiates 12 virotypes (A, B, C1, C2, C3, D1, D2, D3, D4, D5, E, and F) (**Supplementary Tables S7**, **S8**). Based on the characterization explained above, the identification of the high-risk lineages of *E. coli* was assigned according to the review of Manges et al. (2019), which defines the global 20 extraintestinal pathogenic *E. coli* (ExPEC) lineages, together with studies applying a comparable typification approach (Yamaji et al., 2018a, 2018b; Díaz-Jiménez et al., 2020).

### 2.7 Whole genome sequencing

ESBL-producing isolates belonging to CC131 were further investigated by whole genome sequencing (WGS). Briefly, DNA was extracted with the DNeasey Blood & Tissue Kit (Qiagen, Hilen, Germany) according to the manufacturer’s instructions. After extraction, the DNA was quantified by an Invitrogen Qubit fluorimeter (Thermo Fisher Scientific, Massachusetts) and assessed for purity using a NanoDrop ND-1000 (Thermo Fisher Scientific, Massachusetts). The genomic DNA libraries for sequencing were prepared using the Nextera XT Library Prep kit (Illumina, CA, USA) according to the manufacturer’s recommendation. Libraries were purified using the Mag-Bind RXN Pure Plus magnetic beads (Omega Biotek), following the instructions provided by the manufacturer. Then, libraries were pooled in equimolar amounts according to the quantification data provided by the Qubit dsDNA HS Assay (Thermo Fisher Scientific). Lastly, the libraries were sequenced in an Illumina NovaSeq PE150 platform, obtaining 100–150 bp paired-end reads, which were trimmed (Trim Galore 0.6.0) and filtered according to quality criteria (FastQC 0.11.9). The quality-filtered reads were assembled *de novo* using Unicycler (v0.4.8) (Wick et al., 2017), which uses an adapted SPAdes (v3.14.0) assembling algorithm (Bankevich et al., 2012). For the comprehensive typing of the isolates, the assembled contigs were analyzed using different bioinformatics tools available at the CGE webpage as specified and applying the thresholds suggested by default when required (minimum identity of 90% and coverage of 60%): SeroTypeFinder 2.0 (Joensen et al., 2015), CHTyper 1.0 (Roer et al., 2018), MobileElementFinder 1.03 (Johansson et al., 2021), PlasmidFinder 2.1, and pMLST 2.0 (Carattoli et al., 2014). For the phylogenetic typing, two different MLST schemes were applied: *E. coli* #1 (Wirth et al., 2006) and *E. coli* #2 (Jaureguy et al., 2008). In addition to this typing, lineage-specific gene markers based on the sequence of CRISPRCasFinder software (https://crisprcas.i2bc.paris-saclay.fr/) were used to identify and type CRISPR and Cas systems within the genomes. ResFinder 4.1 was used for the identification of acquired genes and/or chromosomal mutations mediating antimicrobial resistance (Camacho et al., 2009; Zankari et al., 2017; Bortolaia et al., 2020). The identification of acquired virulence genes was performed using the web-based tool VirulenceFinder 2.0 (Joensen et al., 2014; Tetzschner et al., 2020). In addition, the bacteria’s pathogenicity towards human hosts was predicted through PathogenFinder 1.1 (Cosentino et al., 2013). Finally, the CSI phylogeny 1.4 tool (Call SNPs & Infer Phylogeny) was used to call, filter and infer a phylogeny based on the concatenated alignment of high-quality single-nucleotide polymorphisms (SNPs) within the core genome.

### 2.8 Statistical analysis

Comparisons of proportions were tested using a two-tailed Fisher’s exact test. The *p*-values < 0.05 were considered statistically significant.

## 3 Results

### 3.1 Phylogeny, PCR screening of virulence traits and non-lactose fermenting isolates

Most of the cUTI isolates analyzed here belonged to the phylogroup B2 (111 of 154; 72.1%), and in turn, most B2 met the UPEC status (107 of 111; 96.4%). On the contrary, only 4 of the 43 (9.3%) non-B2 isolates (*p*-value <0.05) showed carriage of ≥3 of the virulence traits genes associated with a higher efficiency in the colonization of the urinary tract (≥3 of *chuA*, *fyuA*, *vat*, and *yfcV* genes) (Spurbeck et al., 2012). The 43 non-B2 isolates showed six different phylogroups: B1 (14; 9.1%); D (9; 5.9%), A (8; 5.19%), E (6; 3.9%), C (4; 2.6%), and F (2; 1.3%).

The PCR screening of *rfb*O25 and *fli*C_H4_ for the presumptive detection of the pandemic ST131 clonal group was positive in 30 and 29 isolates out of 154, respectively. Additionally, the flagellar-encoding gene *fli*C_H5_, typically associated with NLF *E. coli* ST1193 or ST131 (CH40-41), was determined in 15 of the 24 NLF *E. coli* (24 of 154; 15.6%) (**Supplementary Table S1**).

### 3.2 Antimicrobial susceptibility testing and genotypic characterization of ESBL genes

**Figure 1:**
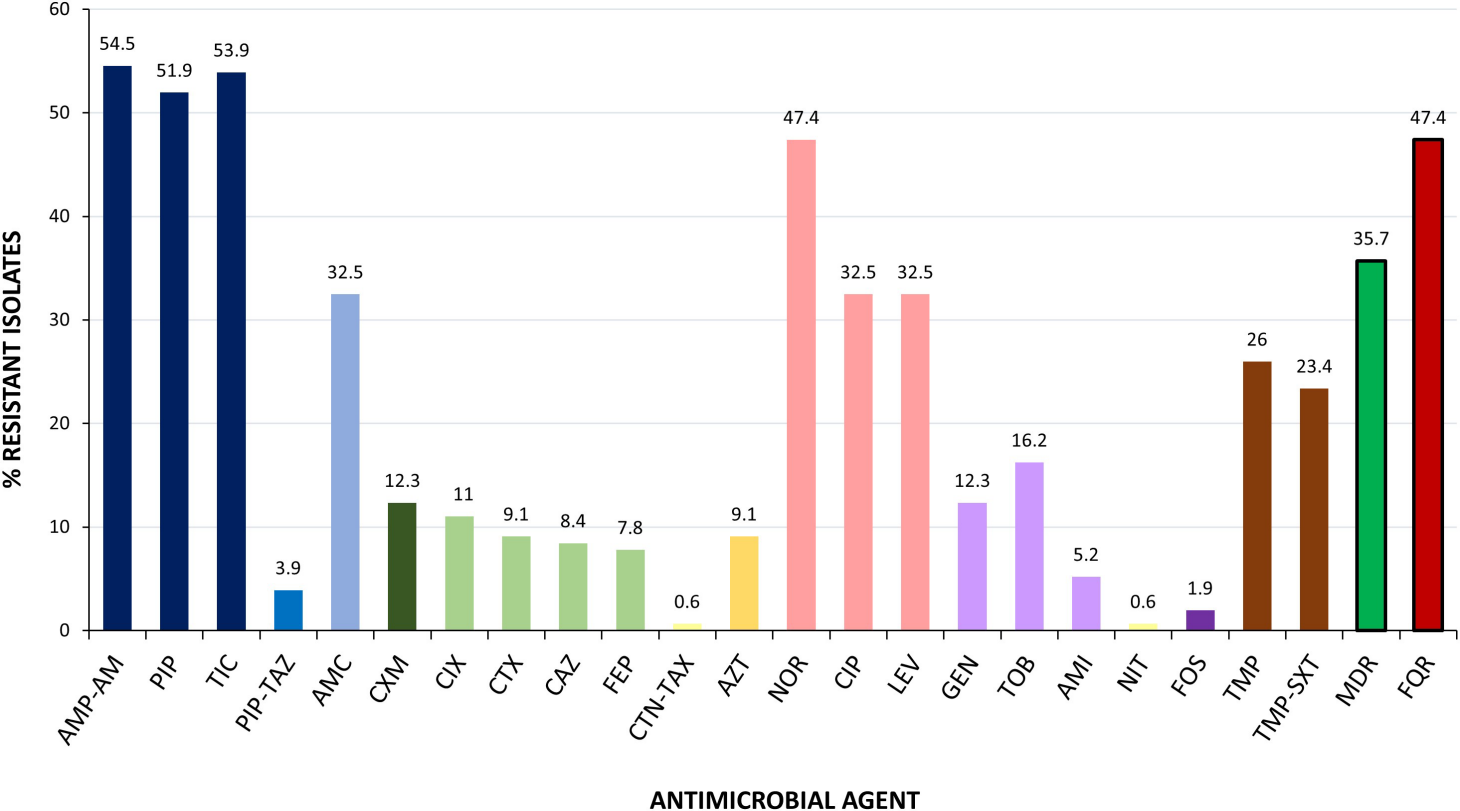
Prevalence of antimicrobial resistance determined among the 154 cUTI *E. coli* analyzed in this study. MICs were interpreted according to EUCAST 2022 breakpoints. AMP-AM, ampicillin/amoxicillin; PIP, piperacillin; TIC, ticarcillin; PIP-TAZ, piperacillin/tazobactam; AMC, amoxicillin/clavulanic acid; CXM, cefuroxime; CIX, cefixime; CTX, cefotaxime; CAZ, ceftazidime; FEP, cefepime; CTN-TAZ, ceftolozane/tazobactam; AZT, aztreonam; NOR, norfloxacin; CIP, ciprofloxacin; LEV, levofloxacin; GEN, gentamicin; TOB, tobramycin; AMI, amikacin; NIT, nitrofurantoin; FOS, fosfomycin; TMP, trimethoprim; TMP-SXT, trimethoprim-sulfamethoxazole; MDR, multidrug resistance; FQR, fluoroquinolone resistance.

The antimicrobial susceptibility testing (AST) of the 154 cUTI isolates showed the highest rates of resistance to ampicillin/amoxicillin (54.5%), ticarcillin (53.9%), and piperacillin (51.9%), followed by norfloxacin (47.4%), ciprofloxacin (32.5%), levofloxacin (32.5%), and amoxicillin-clavulanic acid (32.5%). Four isolates were categorized as colistin resistant by the MicroScan System; however, they were classified as susceptible by the standard broth microdilution (BMD) method (MICs < 0.25 mg/L). The PCR screening of *mcr* genes in those isolates gave a negative result, as well. Globally, 55 of the 154 cUTI (35.7%) isolates showed MDR, being *in vitro* resistant to ≥3 antimicrobial categories (**Figure 1**; **Supplementary Table S1**). Isolates phenotypically suspected of being extended-spectrum cephalosporin resistant were further investigated by PCR for the presence of *bla*_ESBL/AmpC_ genes. As a result, *bla*_CTX-M-15_ and *bla*_CIT_ were determined in 12 and 1 isolates, respectively (**Supplementary Table S1**). Although the group of cUTI isolates positive for the UPEC status exhibited lower prevalence of MDR (38 of 111, 34.2%), the difference was not statistically significant in comparison with those negative for the UPEC status (17 of 43, 39.5%) (*p-*value = 0.58). It is important to note that most of the FQR isolates (52 of 73; 71.2%) exhibited status UPEC; besides, 46 (63%) of 73 were MDR.

### 3.3 Clonal groups and workflow for the surveillance of fluoroquinolone resistance

**Figure 2:**
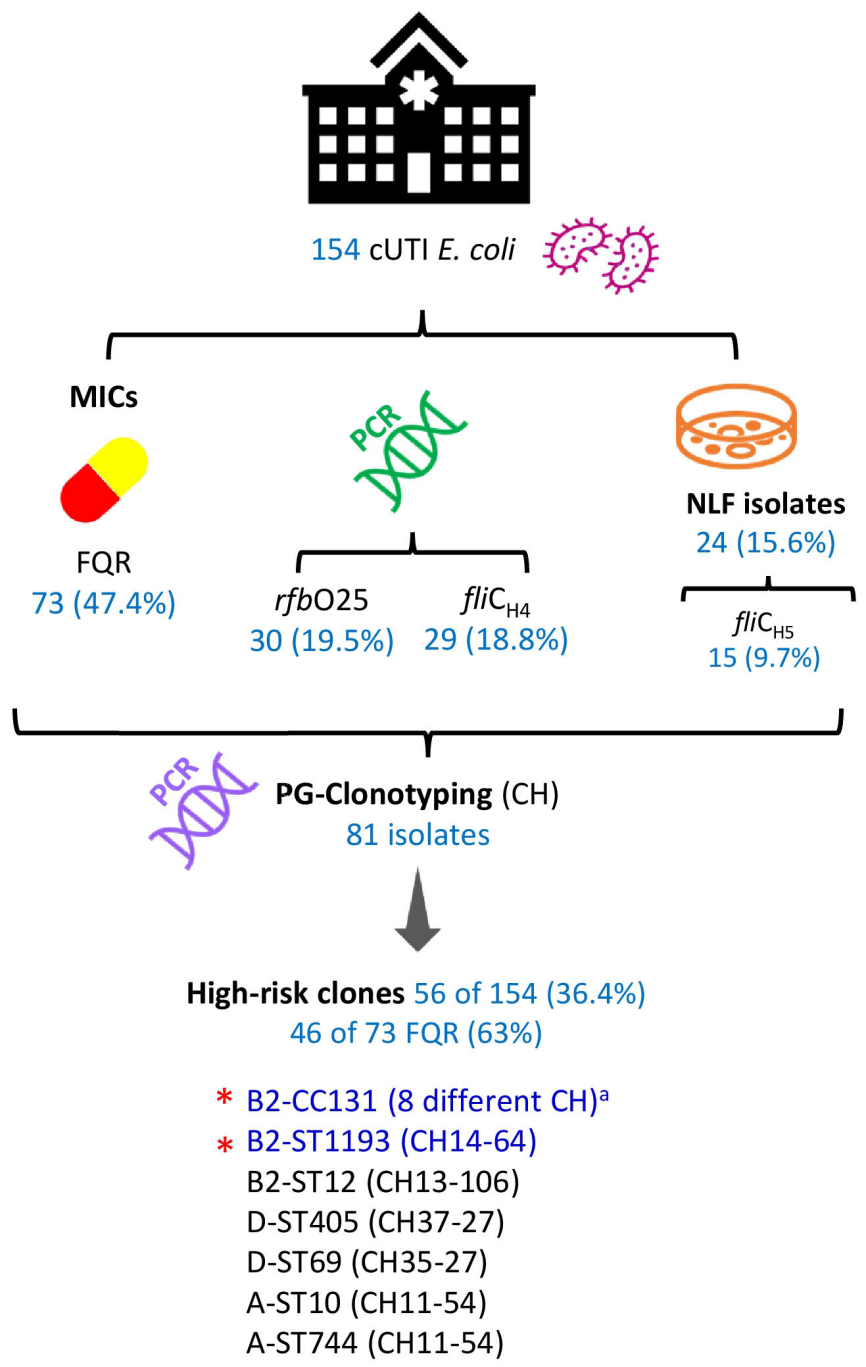
Workflow for the surveillance of prevalent high-risk FQR clones and main results of the 154 cUTI *E. coli* analyzed in this study. cUTI, complicated urinary tract infection; MICs, minimal inhibitory concentrations; FQR, fluoroquinolone resistance; NLF, non-fermenting lactose; PG, phylogroup. ^a^ CH40-30, CH40-41, CH40-22, CH1267-30, CH40-27, CH40-331, CH40-418, and CH40-602. *The most prevalent, representing 28% of the 154 cUTI *E. coli* and 52% of the 73 FQR isolates.

A total of 81 out 154 cUTI *E. coli* were investigated for their clonotypes. The selection criterion for the 81 was to include isolates positive for any of the following traits associated with high-risk clones: FQR, carriers of *rfb*O25 and *fli*C_H4_ genes, or being positive for H5-NLF. As a result, 29 different *fum*C-*fim*H (CH) combinations were established. Nevertheless, 10 CH combinations comprised 70.4% of the 81 isolates, which were fully characterized by MLST (**Table 1**; **Supplementary Table S1**). The main finding here is that both the pandemic clonal complex B2-CC131 and clonal group B2-ST1193 (CH14-64) comprised 28% of the 154 cUTI *E. coli* (33 and 10 isolates, respectively), and most importantly, they represented 52.1% of the FQR isolates. Other prevalent FQR clonal groups were D-ST69 (CH35-27), D-ST405 (CH37-27), and B2-ST429 (CH40-20), determined in three isolates each (**Table 1**). **Figure 2** summarizes the workflow suggested for the surveillance of prevalent high-risk FQR clones and the main results obtained here.

**Table 1:** Prevalent clonotypes (≥3 isolates) within the 81 cUTI isolates positive for any of the surveillance traits proposed here, including FQR, O25b:H4, and H5-NLF isolates.

Clonotype (CH)	Phylogroup-ST (CC)	No. isolates (N=57)	UPEC status (N=44)	Non-lactose fermenting (N=21)	FQR (N=51)	MDR (N=32)
CH11-54	A-ST10 (CC10)	2	0	0	2	1
	A-ST744 (CC10)	1	0	0	1	0
CH65-32	B1-ST162 (CC469)	2	0	1	2	2
	B1-ST1431 (NONE)	2	0	0	2	0
CH40-20	B2-ST429 (CC429)	3	3	1	3	0
CH40-30	B2-ST131^a^ (CC131)	19	19	3	16	12
CH40-22		4	4	0	3	0
CH40-41		4	4	2	4	2
CH14-64	B2-ST1193 (CC14)	10	9	10	10	8
CH13-106	B2-ST12 (CC12)	4	4	4	2	1
CH37-27	D-ST405 (CC405)	3	0	0	3	3
CH35-27	D-ST69 (CC69)	3	1	0	3	3

a In addition to ST131, the clonal group CC131 also appeared to be associated with ST9126. In total, the 33 CC131 isolates exhibited eight clonotypes: CH40-30 (19 isolates/16 FQR), CH40-41 (four isolates/four FQR), CH40-22 (four isolates/three FQR), CH1267-30 (two isolates/two FQR), CH40-27 (one isolate/zero FQR), CH40-331 (one isolate/one FQR), CH40-418 (one isolate/one FQR), and CH40-602 (one isolate/one FQR). UPEC, uropathogenic *E. coli*; FQR, fluoroquinolone resistant; MDR, multidrug resistant.

As expected, all *E. coli* of ST1193 were NLF and also other 14 isolates, including the four representants of the high-risk clonal group B2-ST12 (CH13-106), or the B2-ST131 (CH40-30) and B2-ST131 (CH40-41) (three and two isolates, each respectively). It is also important to highlight the high diversity found within the 33 CC131 isolates, in terms of STs (131 and 9126) and clonotypes: CH40-30 (19 isolates/16 FQR), CH40-41 (four isolates/four FQR), CH40-22 (four isolates/three FQR), CH1267-30 (two isolates/two FQR), CH40-27 (one isolate/zero FQR), CH40-331 (one isolate/one FQR), CH40-418 (one isolate/one FQR), and CH40-602 (one isolate/one FQR) (**Table 1**). Except for the 4 ST131 (CH40-41) isolates, the remaining 29 CC131 were positive for *rfb*O25 and *fli*C_H4_. Finally, the *fli*C_H5_ was determined in all ST1193 isolates, in two out of four ST12, three of four ST131 (CH40-41), and one belonging to B2-ST404 (CH14-27) (**Supplementary Table S1**).

### 3.4 Molecular and *in silico* characterization of CC131 isolates

**Figure 3:**
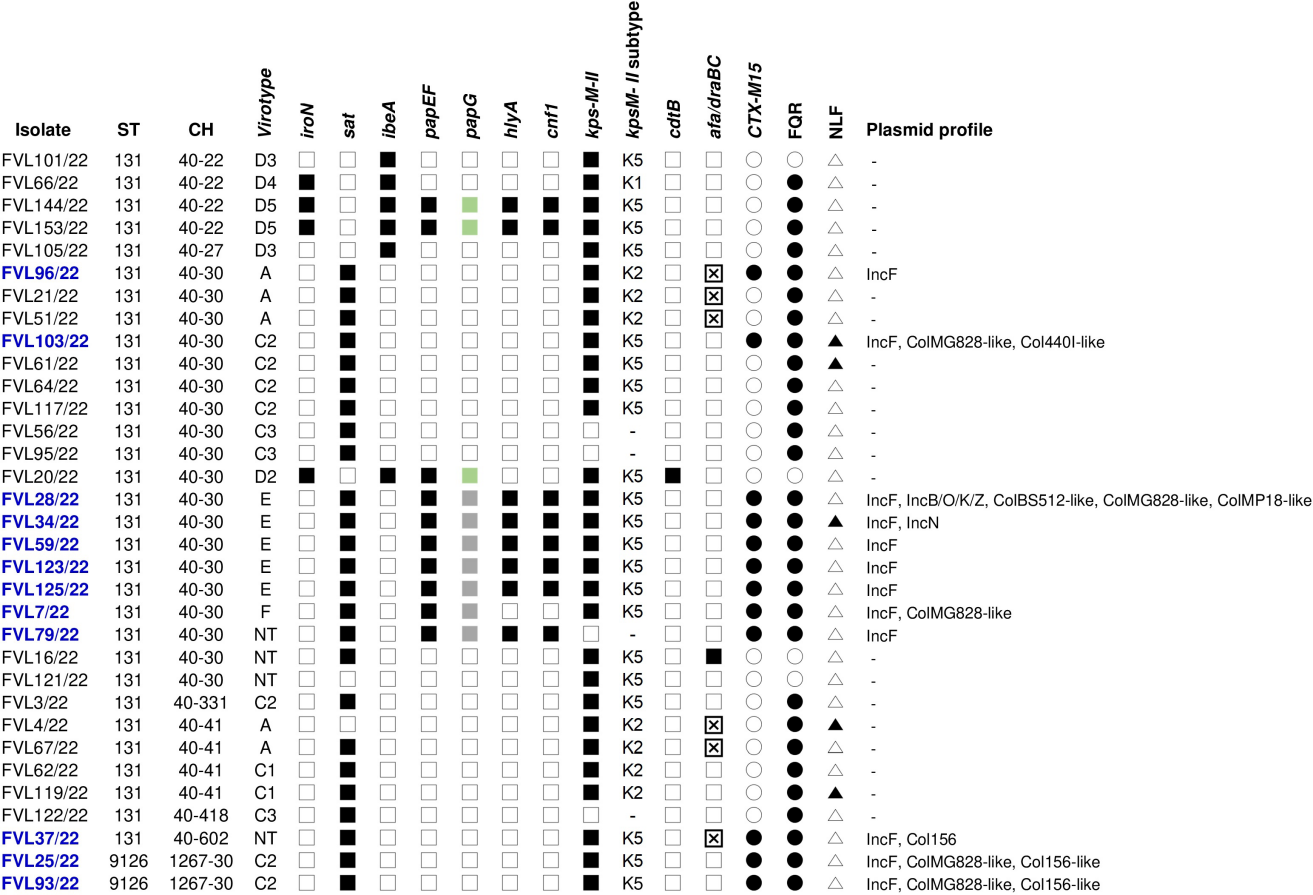
Schematic representation of the 33 CC131 isolates identified in this study and characterized for: sequence type (ST), clonotype (CH), virotype, ESBL typing, fluoroquinolone resistance (FQR), lactose fermenting (non-lactose fermenting: NLF), and plasmid profile. The black and white shading indicate the presence or absence of the corresponding trait, respectively. In blue, isolates further analyzed by WGS; 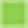 presence of allele III of *pap*G gene, 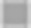 presence of allele II of *pap*G gene, 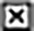 presence of Afa/Dr adhesins (*afa/draBC*) and *afa* operon FM955459, – not determined.

**Figure 4:**
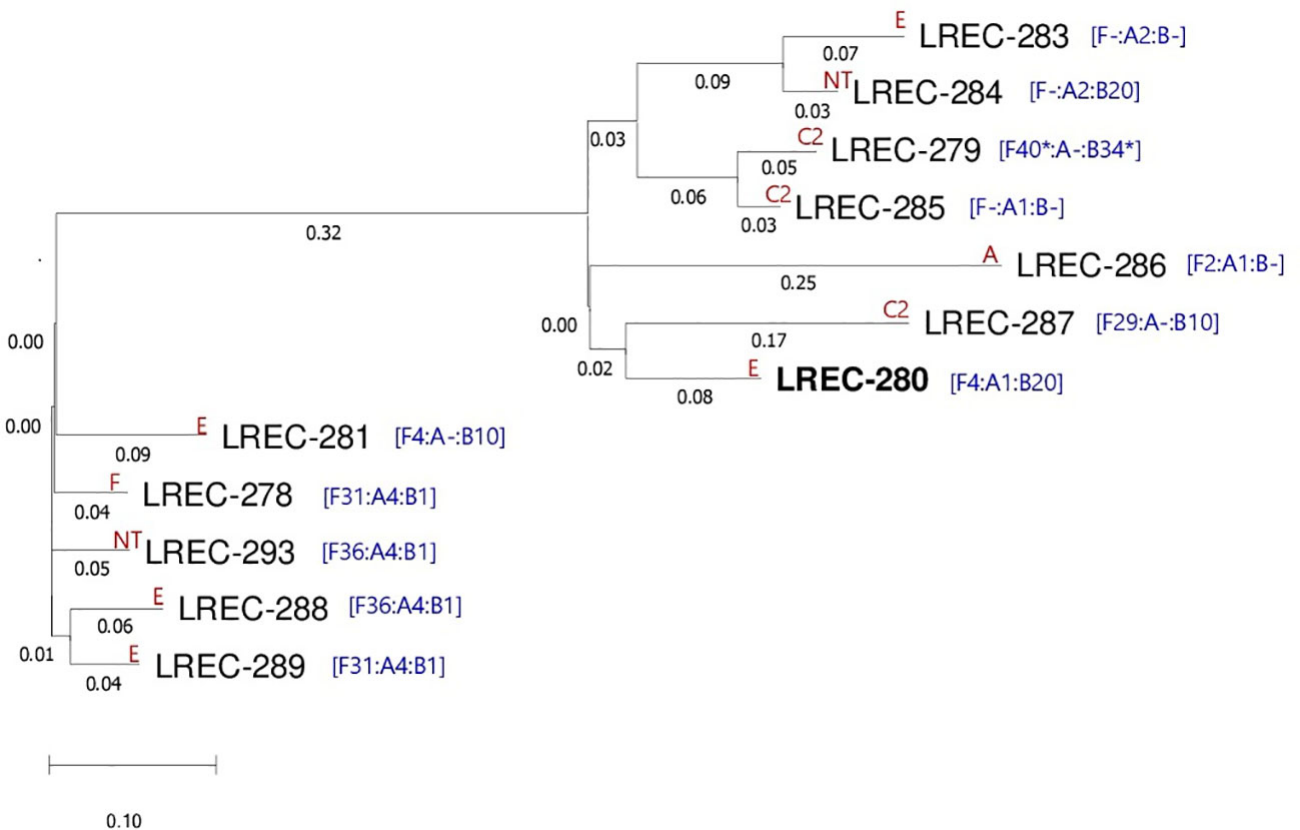
Phylogenetic dendrogram based on the SNP counts per substitution within the core genome of the 12 ESBL ST131 isolates. The comparison of the WGS data sets resulting in a core genome represented by 81.43% of the reference genome LREC-280 (size, 5.4Mb). The tree was constructed with the CSI phylogeny version 1.4 (CGE, https://cge.cbs.dtu.dk/services/CSIPhylogeny/; parameters used for phylogenetic analysis: min. depth at SNP positions ×10; min. relative depth at SNP positions: ×10; min. distance between SNPs (prune): 10 bp; min. SNP quality: 30; min. read mapping quality: 25, a min. Z-score of 1.96 and by ignoring heterozygous SNPs). Values at each branch show the substitutions/site. The reference genome is highlighted in bold. The virotypes and IncF formulae of each isolate are indicated in red and blue, respectively.

The CC131 isolates were molecularly characterized for the presence of specific virulence traits, whose result further corroborated the diversity within the clonal complex. Thus, 10 different virotypes were assigned to the 33 CC131: virotype C2 (seven isolates); virotypes A and E (five isolates each); virotype C3 (three isolates); virotypes C1, D3, and D5 (two isolates each); and virotypes D2, D4, and F (one isolate each). Additionally, the virulence profile of four CC131 remained non-typeable (NT). Of the 15 specific extraintestinal virulence factors included in the scheme of Dahbi et al. (2014), the secreted autotransporter toxin (*sat*), the K5 group II capsule (*kps*M II-K5), and the P fimbriae associated with pyelonephritis together with the specific pilus tip adhesin molecule (*pap*EF and *pap*G) were the most prevalent (76%, 64%, and 30%, respectively). The co-occurrence of *pap*G, *cnf*1 (cytotoxic necrotizing factor 1), and *hly*A (α-hemolysin) in eight isolates (24%) is also outstanding (**Supplementary Table S8**). **Figure 3** represents the main traits of the 33 CC131 isolates, which mostly showed FQR (87.9%), and 12 (36.4%) were additionally ESBL-producers (CTX-M-15). The latter were whole genome sequenced and *in silico* analyzed using different bioinformatics tools of the Center for Genomic Epidemiology (CGE) (**Supplementary Table S9**). Thus, the SerotypeFinder tool confirmed O25b:H4 in all genomes. The 12 exhibited ST43, predicted by means of the alternative MLST scheme (ST#2) based on eight genes (*dinB*, *icdA*, *pabB*, *polB*, *putP*, *trpA*, *trpB*, and *uidA*). However, the MLST seven-gene scheme (ST#1) discriminated two STs (131 and 9126), and the core genome multi-locus typing (cgMLST) of 2,512 loci, differentiated the following 10 cgSTs: 7829 (for the two ST9126 isolates); 29126 (for two ST131 CH40-30 isolates); and 12614, 142625, 116708, 139233, 122338, 85838, 71301, and 13547 (one isolate, each). Regarding the resistome, the presence of *bla*_CTX-M-15_ gene was confirmed in the 12 CC131 genomes, which also showed the same set of chromosomal mutations conferring FQR (*gyrA* p.S83L, *gyrA* p.D87N, *parC* p.S80I, *parC* p.E84V, and *parE* p.I529L), consistent with the *in vitro* expression. As shown in **Supplementary Table S9**, most of the resistance genes were bracketed by different mobile genetic elements (MGEs), mainly insertion sequences, such as *ISEc*9, which was found with *bla*_CTX-M-15_ gene in six genomes. Furthermore, a wide range of extraintestinal virulence genes were predicted in the 12 genomes. The prediction of F4 (K88) fimbrial encoding genes *fae*C, *fae*D, *fae*F, *fae*H, and *fae*l associated with an *IS*640 on contig 23 of LREC-279 (ST9126) is outstanding because this is a typical fimbrial antigen of porcine enterotoxigenic *E. coli* (García-Meniño et al., 2021). Globally, the plasmidome analysis revealed not only the prevalent presence of IncF plasmids but also the high diversity of pMLST types represented by 10 different IncF formulae. In addition, six genomes showed carriage of small Col-like plasmids. The 12 ST131 genomes were predicted as human pathogen (probability >92%) by the PathogenFinder tool of the CGE. The genomic similarity of the 12 assemblies were further investigated through the SNP comparison of their core genome represented by 81.43% of the reference genome LREC-280 (size, 5.4Mb) using CSI phylogeny 1.4 (**Figure 4**; **Supplementary Table S10**). The phylogenetic dendrogram showed different subclusters. As expected, LREC-279 and LRC-285 (ST9126 and cgST7829) clustered with only 75 SNP differences. Likewise, the two cgST29126 (LREC-283 and LREC-284) showed 105 SNP differences. On the contrary, the LREC-288 exhibited the maximum distance of 487 SNPs with LREC-286.

## 4 Discussion

It is broadly accepted that UTIs are one of the most common and widespread community-acquired and nosocomial infections worldwide (Klein and Hultgren, 2020), constituting one of the leading causes of antibiotic prescriptions (Aabenhus et al., 2017). In addition, UTIs are also adding to the public health burden of infectious-related deaths, as recently reported in the systematic analysis for the Global Burden of Disease Study 2019 (Ikuta et al., 2022). Unfortunately, there are still no global harmonized protocols for the systematically monitoring of emerging successful FQ and cephalosporin-resistant clones implicated in this complex group of infections, which help to maintain the effectiveness of available treatment options.

Following our previous proposal on uUTI, based on a clonal diagnosis approach jointly with AST and the screening of predictor genes of uropathogenicity (García-Meniño et al., 2022), we addressed here the characterization of 154 non-duplicated *E. coli* recovered as the only pathogen involved in cases of cUTI. Our aim was to compare both syndromes in terms of the *E. coli* involved and to improve the surveillance protocol based on new observations. As a result, we found a similar phylogenetic distribution of the *E. coli* population implicated in uUTI or cUTI, being B2 the predominant phylogroup (67% and 72.1%, respectively), in line with its worldwide predominance in UTIs (Flament-Simon et al., 2020b; Lin et al., 2021). We also found a similar UPEC status prevalence within B2 (97% and 96.4%, respectively). No significant difference in the level of MDR was observed, either (30% vs. 35.7%); however, the AST showed important differences regarding the FQR (28% of uUTI vs. 47.4% of cUTI isolates; *p* = 0.002) and presence of ESBL producers (0% isolates in uUTI vs. 7.8% in cUTI; *p* = 0.002). Looking into the FQR clones implicated in UTI, we found a wide diversity of PG-CH combinations, some of them shared in both uUTI and cUTI, such as A (CH11-54), B1 (CH65-32), B2 (CH14-64), B2 (CH38-15), B2 (CH40-41), or D (CH37-27). While it is relevant that the FQR clone B2-ST1193 (CH14-64) exhibited similar implication in both studies (6% in uUTI and 6.5% in cUTI), it is also of note the higher prevalence and genetic diversity of the CC131 in cUTI isolates (33, 21.4%), from which 28 were FQR (18.2%) including 12 ESBL-producers (7.8%), vs. 6% CC131 in uUTI and only 2% FQR (García-Meniño et al., 2022).

MDR UPEC infections are globally associated with the major pandemic clonal lineages ST131 (CH40-H30*R*) and ST1193 (CH14-64), as the first and second most prevalent clones among FQ/cephalosporin-resistant *E. coli*, respectively (Colpan et al., 2013; Banerjee and Johnson, 2014; Pitout et al., 2022). Different studies assessed the success of the ST131 as a dominant pandemic ExPEC, linked with ESBL-production and/or FQR (Banerjee and Johnson, 2014) and even, in some cases, with the carriage of carbapenemase- or colistin-resistance-encoding genes (Peirano et al., 2014; García-Meniño et al., 2018; Taati Moghadam et al., 2021). The clone B2-ST1193, with the O type 75, is another example of successful ExPEC. However, and despite the fact that it is already reported as an important contributor to the FQR across several countries, i.e., France (Birgy et al., 2020), Germany (Valenza et al., 2019), China (Ding et al., 2021), Korea (Kim et al., 2017), Vietnam (Nguyen et al., 2021), and the United States (Tchesnokova et al., 2019), it has been rarely identified in Spain (Flament-Simon et al., 2020b; García-Meniño et al., 2022; Becerra-Aparicio et al., 2023). Of note, the ST1193 isolates of the present study were recovered from patients between 38 and 85 years old. Here, the ST1193 was not associated with ESBL production.

Regarding the ST131 pandemic lineage, its evolution over time is represented by three main clades (susceptible clades A and B and FQ/cephalosporin-resistant clade C), associated with different alleles of the fimbrial adhesion type 1 (*fim*H). The clade C (*fim*H30, clonotype CH40-30) is the most widely disseminated worldwide (Pitout and DeVinney, 2017). The CH40-30 FQR non-ESBL-producing isolates are assigned to the subclade C1 (H30*R*), and those that, in addition to the FQR traits, also harbor the *bla*_CTX-M-15_ are classified within the subclade C2 (H30*Rx*). They are statistically associated with recurrent or persistent UTI and sepsis, and some authors have suggested that their success could be linked with their capacity of adaptation and sequential acquisition of virulence factors, FQR, and ESBL production, in a context in which the use of FQ and cephalosporins is increasing globally (Banerjee and Johnson, 2014). According to the *fim*H type, the 31 ST131 of our study would be assigned to clades A (*fim*H41, four isolates/four FQR), clade B (*fim*H22, four isolates/three FQR), and clade C (*fim*H30, 19 isolates/16 FQR/9 CTX-M15). The remaining ST131 possessed *fim*H27 (one non-FQR isolate) and *fim*H331, *fim*H418, and *fim*H602 (one FQR isolate each). The age range of the patients is striking, namely, between <1 year and 97 years old. Specifically, the two youngest, 8 months and 2 years old, presented isolates of the clonotypes CH40-30 and CH40-41 (FQR). The CC131 here was additionally represented by another clone, B2-ST9126 (CH1267-30), which is a very rare ST. In fact, there are only seven genomes uploaded in the Enterobase repository (as of November 2023; https://enterobase.warwick.ac.uk/), whose metadata refers to human origin, recovered in USA, Canada, and Spain (the two of the present study belonging to different cgST). Our ST9126 isolates were from two women of 64 and 87 years old.

On the other hand, it is well described that the evolution of the C1 and C2 subclades was strongly influenced by the acquisition and replacement of the specific plasmids, F1:A2:B20 and F2:A1:B-, that are predominant in those C1 (H30R) and C2 (H30Rx) subclades, respectively (Johnson et al., 2016; Mahérault et al., 2019; Kondratyeva et al., 2020). Interestingly, we observed here a high diversity within the nine sequenced members of CC131-H30*Rx* subclade, carriers of different plasmid STs (F31:A4:B1, F4:A1:B20, F4:A-:B10, F-:A2:B-, F-:A2:B20, F2:A1:B-, F29:A-:B10, F36:A4:B1, and F31:A4:B1), where only one STS131 (CH40-30) showed the carriage of the predominant F2:A1:B- plasmid type. Taking as a reference previous studies/reviews (Nicolas-Chanoine et al., 2014), including those performed in our north-west health area of Spain (Mamani et al., 2019; Flament-Simon et al., 2020b; García-Meniño et al., 2022), it seems that a phylogenetic diversification within CC131 is currently accelerating. We could hypothesize the different origin of plasmid acquisition of these CC131 implicated in cUTI; however, this assertion requires broader and deeper genomic analyses.

Previous studies performed in our country found different ESBL-producing *E. coli* prevalences in cUTIs. In a multicenter study, Merino et al. (2016) analyzed bacteriemia episodes associated with UTIs (n = 425) from eight hospitals from different Spanish geographical areas (2010–2011), and in a later work, Flament-Simon et al. (2020b) studied 100 non-duplicate *E. coli* consecutively obtained from different clinical samples, but mostly UTIs happened during 2016 in Spain. These authors reported a prevalence of 9.2% (39/425) and 6% (6/100) of ESBL-producing *E. coli*, respectively, which are similar values to the prevalence detected here (7.8%). Conversely, Becerra-Aparicio et al. (2023) reported a significant higher value (30.6%; 68/222) in *E. coli* isolates recovered from patients with healthcare-associated bacteremia of urinary origin from 12 Spanish tertiary hospitals (2017–2019). In agreement with our findings, most of the ESBL-producing isolates involved in UTIs, here and other countries, are associated with the pandemic ST131. Also in Spain, Flament-Simon et al. (2020a) found that 40.5% of 84 non-duplicated ESBL-producing *E. coli* causing UTIs were assigned to the CC131. This tendency was also reported by Merino et al. (2016) and Becerra-Aparicio et al. (2023), with prevalences of 54% (21/39) and 67.6% (46/68) of the ESBL-producing isolates assigned to ST131, respectively.

A difference found in our health area in comparison with other studies in Spain or other countries is regarding the subclade C1-M27 of ST131, which seems to be displacing the clade C2/CTX-M-15 in certain parts of the world, especially in Japan and Europe (Peirano and Pitout, 2019; Becerra-Aparicio et al., 2023). Our results suggest that C1-M27 is not yet disseminated in the health area of Oviedo, and the *bla*_CTX-M-15_ is still the predominant *bla* gene in Spain. Specifically, Flament-Simon et al. (2020a) reported a prevalence of 82.3% (n: 28/34) for the *bla*_CTX-M-15_ gene and 14.7% (n: 5/34) for the *bla*_CTX-M-27_. In the study of Becerra-Aparicio et al. (2023), most of the isolates assigned to the C2/H30*Rx* subclone harbored the CTX-M-15 (37/44), and four belonged to the subclade C1-M27 (*bla*_CTX-M-27_). All the divergences abovementioned with respect to the prevalence of the CC131 and ESBL-producing *E. coli* in Spain can be due to different factors, namely, i) a longer and higher exposition to last-line antibiotics of patients with healthcare-associated bacteremia of urinary origin compared to those that have not yet developed an invasive infection and ii) the specific epidemiological context in which the study is taking place.

In the guidelines on urological infections of the European Association of Urology (EAU) (European Association of Urology, 2023), there are specific recommendations for the treatment of cUTIs. Thus, the EAU recommends as a first-line therapy i) the amoxicillin in combination with aminoglycosides, ii) a second-generation cephalosporin also in combination with aminoglycosides, or iii) a third-generation cephalosporin intravenously in the case of patients with systemic symptoms. FQs are not automatically suitable as empirical antimicrobial therapy for cUTI. In fact, ciprofloxacin can be provided if the local resistance percentages are <10% only under certain circumstances (entire treatment given orally, in patients that do not require hospitalization or in patient that has an anaphylaxis for beta-lactam antimicrobials). Finally, ciprofloxacin cannot be used as an empirical treatment in patients from urology departments or when patients have used fluoroquinolones in the last 6 months. The AST results obtained here with antimicrobial resistance prevalences of 54.5% ampicillin/amoxicillin, 12.3% gentamicin, 16.2% tobramycin, 5.2% amikacin, 12.3% cefuroxime, 8%–9% third-generation cephalosporin and 32.5-47.4% FQ, mean a serious therapeutic compromise for more than 10% of patients.

Specifically, the FQR found here is of great concern. FQ are included in the list of critically important antimicrobials for human medicine of the World Health Organization (WHO). The WHO also reports the worrisome prevalence of FQR among *E. coli* and global dissemination of FQR determinants within environmental, commensal, and pathogenic organisms (WHO, 2014; ECDC, 2022). The latest report from EFSA/ECDC (EFSA/ECDC, 2023) and recent studies indicate an increasing prevalence of FQR *E. coli* isolates in livestock animals, specifically among Shiga toxin–producing *E. coli* (STEC) and enterotoxigenic *E. coli* (ETEC) causing diarrhea in pigs increasing from 56.5% in 2005–2017 (García-Meniño et al., 2021) to reach up to 77% in the period 2020–2022 (García et al., 2022). Going beyond, we also investigated food-borne transmission of potentially UPEC in a parallel study (same period, 2020, and same health area, Oviedo), with a representative sampling of meat (pork, beef, chicken, and turkey) in different supermarkets. We found that 18% of the samples were carriers of *E. coli* FQR and 38% of ESBL-producing *E. coli*. Furthermore, we detected that 6% of the meat samples were carriers of *E. coli* positive for the status UPEC. Some of the meat isolates were further assayed in human bladder cells to prove a similar *in vitro* behavior for certain *E. coli* clones of animal origin positive for the UPEC status, compared to human UTI isolates, reinforcing the role of food-producing animals as a potential source of UPEC for consumers (García et al., 2023). This kind of studies, using the One Health approach, are a priority to understand the flow within the different hosts and ecosystems and to implement control measures.

In UPEC, resistance to FQ is significantly higher in the hospital setting in comparison with the community (Fasugba et al., 2015) and in developing countries (55.5%–85.5%) with respect to that in developed countries (5.1%–32.0%) (Kot, 2019). Specifically, in a multicenter study performed between 2013 and 2014 in Belgium, Germany, and Spain, the percentage of ciprofloxacin-resistant UPEC strains was 12.9%, 17.3%, and 39.8%, respectively (Kresken et al., 2016). Another study conducted in England showed that the rate of ciprofloxacin-resistant *E. coli* obtained from urine samples ranged between 15.5% and 20.4% (Abernethy et al., 2017). In the United States (2013–2014), 12.1% of the *E. coli* isolates from patients with acute uncomplicated and complicated pyelonephritis was resistant to ciprofloxacin (Talan et al., 2016), and a 5.1% was detected between 2016 and 2017 from the urine samples of patients with UTI (Yamaji et al., 2018b). As mentioned before, in our collection, the rate of resistance against different FQ is remarkably higher in comparison with these studies.

Mutations in several genes targeting serine residues in DNA gyrase (GyrA-S83L) and topoisomerase IV (ParC-S80I) and a common combination with an overall deleterious mutation in GyrA-D87N can lead to resistance development against FQ. Another mechanism of resistance is the acquisition of specific plasmid-mediated quinolone resistance (PMQR) *qnr* genes, which are commonly located on mobile-resistant elements (Poirel et al., 2005). The emerging lineages ST131-H30*R* and ST1193 are, nowadays, playing the prominent role in the dissemination and maintenance of FQR. In the ST131 genomes here analyzed and in ST1193 genomes of a previously published paper (García-Meniño et al., 2022), the presence of *qnr* genes is scarce (predicted in 1 out of the 12 ST131 genomes; LREC-281) or null, respectively. The transmission of these resistances is then mainly linked to chromosomal point mutations transmitted vertically in these relevant lineages (Price et al., 2013; Johnson et al., 2018), while *qnr* genes are not playing a major role. On the other hand, it is suggested that the sequential acquisition of double serine quinolone-resistant determining region mutations (QRDR) (GyrA-S83L and ParC-S80I), typically presented in these predominant MDR clonal groups of *E. coli* (Pitout et al., 2022), as we proved in our cUTI and uUTI collections, has been probably a key driver behind the success of the pandemic dissemination of these clones in the last few decades (Tchesnokova et al., 2023). Thus, the 12 FQR ST131 sequenced here showed the common set of mutations (*gyrA* p.S83L, *gyrA* p.D87N, *parC* p.S80I, *parC* p.E84V, and *parE* p.I529L), and the ST1193 genomes analyzed in the previous study exhibited the same FQR pattern (*gyrA* p.S83L, *gyrA* p.D87N, *parC* p.S80I, and *parE* p.L416F) (García-Meniño et al., 2022), in both cases correlating with the *in vitro* expression. Furthermore, the QRDR point mutations would be generating a fitness benefit, which would explain why the bacteria keep them. Therefore, the substantial reduction in the prescription of FQ in the recent decade is not significantly generating a decrease in the percentage of FQR or decrease in the circulation of these MDR clones (Tchesnokova et al., 2023). In addition to the energetically favorable QRDR mutations, the carriage of low-cost plasmids and integrons with weak promoters and some virulence factors (colonization-associated genes) may have also a fitness-favorable impact in these clones (Fuzi and Sokurenko, 2023). On the other hand, the need of an appropriate genetic background exposed by the selective epistasis hypothesis would explain why other *E. coli* strains with the same set of QRDR mutation could not achieve the same level of notoriety (Cummins et al., 2021; Fuzi and Sokurenko, 2023). In this context, the spread of highly successful clones that manifest an adaptative advantage with respect to other clones, and with capacity of colonization, currently represents a serious sanitary issue despite a reduction in antibiotic pressure.

## 5 Conclusions

The global MDR high-risk clones of *E. coli* CC131 and ST1193 represent a challenge for the treatment of cUTIs in our health area, since 52% of the FQR isolates analyzed here belonged to these lineages. Although *E. coli* CC131 and ST1193 are also involved in the community uUTIs of this geographic area, the differences in prevalence and resistance background would be explained by a lower exposure to antibiotic treatments. Interventions to eradicate these specific FQR clones, such as the design of vaccines against them, along with surveillance for other emerging ones, are essential for antibiotic use optimization programs. Based on our results, we suggest some key traits for a lab workflow monitoring of cUTIs. Thus, a non-lactose fermenting screening, together with the detection of O25b (*rfb*O25b), H4 (*fliC_H4_*), and H5 (*fliC_H5_*) genes, and phylogroup and clonotyping assignation, is a reasonable approach that can be easily implemented for the surveillance of successful and emerging *E. coli* high-risk clones associated with FQR spread in cUTIs, such as the uncommonly reported O25b:H4-B2-ST9126 (CH1267-30) of the CC131.

## Data availability statement

The datasets presented in this study can be found in online repositories. The names of the repository/repositories and accession number(s) can be found below: https://www.ebi.ac.uk/ena, ERS12564407 to ERS12564418.

## Ethics statement

The studies involving humans were approved by the Comité de Ética de la investigación con medicamentos del Principado de Asturias, Hospital Universitario Central de Asturias (Spain), with code CEImPA 2021.531. The studies were conducted in accordance with the local legislation and institutional requirements. The human samples used in this study were acquired from primarily isolated as part of our previous study for which ethical approval was obtained. Written informed consent for participation was not required from the participants or the participants’ legal guardians/next of kin in accordance with the national legislation and institutional requirements.

## Author contributions

IG-M: Formal analysis, Investigation, Methodology, Software, Validation, Visualization, Writing – original draft. VG: Formal analysis, Methodology, Software, Writing – original draft, Validation, Visualization. PL: Methodology, Software, Validation, Visualization, Writing – original draft. JF: Formal analysis, Funding acquisition, Investigation, Project administration, Validation, Visualization, Writing – review & editing. AM: Conceptualization, Formal analysis, Funding acquisition, Investigation, Project administration, Supervision, Validation, Visualization, Writing – original draft, Writing – review & editing.

## References

[B1] Aabenhus R. Hansen P. Siersma V. Bjerrum L. (2017). Clinical indications for antibiotic use in Danish general practice: results from a nationwide electronic prescription database. Scandinavian J. Primary Health Care 35:2, 162–169. doi: 10.1080/02813432.2017.1333321 PMC5499316 28585886

[B2] Abernethy J. Guy R. Sheridan E. A. Hopkins S. Kiernan M. Wilcox M. H.. (2017). Epidemiology of Escherichia coli bacteraemia in England: results of an enhanced sentinel surveillance programme. J. Hosp. Infect. 95, 365–375. doi: 10.1016/j.jhin.2016.12.008 28190700

[B3] Aydin A. Ahmed K. Zaman I. Khan M. S. Dasgupta P. (2015). Recurrent urinary tract infections in women. Int. Urogynecol. J. 26, 795–804. doi: 10.1007/s00192-014-2569-5 25410372

[B4] Banerjee R. Johnson J. R. (2014). A new clone sweeps clean: the enigmatic emergence of Escherichia coli sequence type 131. Antimicrob. Agents Chemother. 58, 4997–5004. doi: 10.1128/AAC.02824-14 24867985 PMC4135879

[B5] Bankevich A. Nurk S. Antipov D. Gurevich A. A. Dvorkin M. Kulikov A. S.. (2012). SPAdes: A new genome assembly algorithm and its applications to single-cell sequencing. Journal of Computational Biology. 19, 455–477. doi: 10.1089/cmb.2012.0021 22506599 PMC3342519

[B6] Becerra-Aparicio F. Gómez-Zorrilla S. Hernández-García M. Gijón D. Siverio A. Berbel D.. (2023). Significant increase of CTX-M-15-ST131 and emergence of CTX-M-27-ST131 Escherichia coli high-risk clones causing healthcare-associated bacteraemia of urinary origin in Spain (ITUBRAS-2 project). J. Antimicrob. Chemother. 78, 2291–2296. doi: 10.1093/jac/dkad234 37533351

[B7] Bortolaia V. Kaas R. S. Ruppe E. Roberts M. C. Schwarz S. Cattoir V.. (2020). ResFinder 4.0 for predictions of phenotypes from genotypes. J. Antimicrob. Chemother. 75, 3491–3500. doi: 10.1093/jac/dkaa345 32780112 PMC7662176

[B8] Camacho C. Coulouris G. Avagyan V. Ma N. Papadopoulos J. Bealer K.. (2009). BLAST+: architecture and applications. BMC Bioinf. 10, 421. doi: 10.1186/1471-2105-10-421 PMC2803857 20003500

[B9] Carattoli A. Zankari E. García-Fernández A. Voldby Larsen M. Lund O. Villa L.. (2014). In silico detection and typing of plasmids using PlasmidFinder and plasmid multilocus sequence typing. Antimicrob. Agents Chemother. 58, 3895–3903. doi: 10.1128/AAC.02412-14 24777092 PMC4068535

[B10] Clermont O. Christenson J. K. Denamur E. Gordon D. M. (2013). The Clermont Escherichia coli phylo-typing method revisited: Improvement of specificity and detection of new phylo-groups. Environ. Microbiol. Rep. 5, 58–65. doi: 10.1111/1758-2229.12019 23757131

[B11] Clermont O. Dixit O. V. A. Vangchhia B. Condamine B. Dion S. Bridier-Nahmias A.. (2019). Characterization and rapid identification of phylogroup G in Escherichia coli, a lineage with high virulence and antibiotic resistance potential. Environ. Microbiol. 21, 3107–3117. doi: 10.1111/1462-2920.14713 31188527

[B12] Colpan A. Johnston B. Porter S. Clabots C. Anway R. Thao L.. (2013). Escherichia coli sequence type 131 (ST131) subclone H30 as an emergent multidrug-resistant pathogen among US veterans. Clin. Infect. Dis. 57, 1256–1265. doi: 10.1093/cid/cit503 23926176 PMC3792724

[B13] Cosentino S. Voldby Larsen M. Møller Aarestrup F. Lund O. (2013). PathogenFinder–distinguishing friend from foe using bacterial whole genome sequence data. PloS One 8 (10), e77302. doi: 10.1371/annotation/b84e1af7-c127-45c3-be22-76abd977600f 24204795 PMC3810466

[B14] Cummins E. A. Snaith A. E. McNally A. Hall R. J. (2021). The role of potentiating mutations in the evolution of pandemic Escherichia coli clones. Eur. J. Clin. Microbiol. Infect. Dis. doi: 10.1007/s10096-021-04359-3 34787747

[B15] Dahbi G. Mora A. Mamani R. López C. Alonso M. P. Marzoa J.. (2014). Molecular epidemiology and virulence of Escherichia coli O16:H5-ST131: comparison with H30 and H30-Rx subclones of O25b:H4-ST131. Int. J. Med. Microbiol. 304, 1247–1257. doi: 10.1016/j.ijmm.2014.10.002 25455219

[B16] Díaz-Jiménez D. García-Meniño I. Fernández J. García V. Mora A. (2020). Chicken and Turkey meat: Consumer exposure to multidrug-resistant Enterobacteriaceae including mcr-carriers, uropathogenic E. coli and high-risk lineages such as ST131. Int. J. Food Microbiol 331, 108750. doi: 10.1016/j.ijfoodmicro.2020.108750 32559710

[B17] Ding Y. Zhang J. Yao K. Gao W. Wang Y. (2021). Molecular characteristics of the new emerging global clone ST1193 among clinical isolates of Escherichia coli from neonatal invasive infections in China. Eur. J. Clin. Microbiol. Infect. Dis. 40, 833–840. doi: 10.1007/s10096-020-04079-0 33118058

[B18] ECDC (2022). Antimicrobial resistance surveillance in Europe 2022 - 2020 data (Copenhagen: WHO Regional Office for Europe).

[B19] EFSA/ECDC (2023). The European Union Summary Report on Antimicrobial Resistance in zoonotic and indicator bacteria from humans, animals and food in 2020/2021. EFSA J. Eur. Food Saf. Auth. 21 (3), 7867. doi: 10.2903/J.EFSA.2023.7867 PMC9987209 36891283

[B20] European Association of Urology (2023). EAU guidelines on Urological Infections (Arnhem, The Netherlands: Eur. Assoc. Urol).

[B21] Fasugba O. Gardner A. Mitchell B. G. Mnatzaganian G. (2015). Ciprofloxacin resistance in community- and hospital-acquired Escherichia coli urinary tract infections: a systematic review and meta-analysis of observational studies. BMC Infect. Dis. 15, 545. doi: 10.1186/s12879-015-1282-4 26607324 PMC4660780

[B22] Flament-Simon S.-C. García V. Duprilot M. Mayer N. Alonso M. P. García-Meniño I.. (2020a). High prevalence of ST131 subclades C2-H30Rx and C1-M27 among extended-spectrum β-lactamase-producing escherichia coli causing human extraintestinal infections in patients from two hospitals of Spain and France during 2015. Front. Cell. Infect. Microbiol. 10. doi: 10.3389/fcimb.2020.00125 PMC7105571 32266173

[B23] Flament-Simon S.-C. Nicolas-Chanoine M.-H. García V. Duprilot M. Mayer N. Alonso M. P.. (2020b). Clonal Structure, Virulence Factor-encoding Genes and Antibiotic Resistance of Escherichia coli, Causing Urinary Tract Infections and Other Extraintestinal Infections in Humans in Spain and France during 2016. Antibiotics 9, 161. doi: 10.3390/antibiotics9040161 32260467 PMC7235800

[B24] Flores-Mireles A. L. Walker J. N. Caparon M. Hultgren S. J. (2015). Urinary tract infections: epidemiology, mechanisms of infection and treatment options. Nat. Rev. Microbiol. 13, 269. doi: 10.1038/nrmicro3432 25853778 PMC4457377

[B25] Foxman B. (2003). Epidemiology of urinary tract infections: incidence, morbidity, and economic costs. Dis. Mon 49, 53–70. doi: 10.1067/mda.2003.7 12601337

[B26] Foxman B. (2010). The epidemiology of urinary tract infection. Nat. Rev. Urol. 7, 653–660. doi: 10.1038/nrurol.2010.190 21139641

[B27] Foxman B. (2014). Urinary tract infection syndromes: occurrence, recurrence, bacteriology, risk factors, and disease burden. Infect. Dis. Clin. North Am. 28, 1–13. doi: 10.1016/j.idc.2013.09.003 24484571

[B28] Fuzi M. Sokurenko E. (2023). Commensal fitness advantage may contribute to the global dissemination of multidrug-resistant lineages of bacteria-the case of uropathogenic E. coli. Pathog. (Basel Switzerland) 12, 1150. doi: 10.3390/pathogens12091150 PMC10536240 37764958

[B29] García V. García-Meniño I. Gómez V. Jiménez-Orellana M. Méndez A. Aguarón A.. (2022). Mobile colistin resistance (MCR), extended-spectrum beta-lactamase (ESBL) and multidrug resistance monitoring in Escherichia coli (commensal and pathogenic) in pig farming: need of harmonized guidelines and clinical breakpoints. Front. Microbiol. 13. doi: 10.3389/fmicb.2022.1042612 PMC9756432 36532469

[B30] García V. Lestón L. Parga A. García-Meniño I. Fernández J. Otero A.. (2023). Genomics, biofilm formation and infection of bladder epithelial cells in potentially uropathogenic Escherichia coli (UPEC) from animal sources and human urinary tract infections (UTIs) further support food-borne transmission. One Heal. (Amsterdam Netherlands) 16, 100558. doi: 10.1016/j.onehlt.2023.100558 PMC10288081 37363240

[B31] García-Meniño I. García V. Alonso M. P. Blanco J. E. Blanco J. Mora A. (2021). Clones of enterotoxigenic and Shiga toxin-producing Escherichia coli implicated in swine enteric colibacillosis in Spain and rates of antibiotic resistance. Vet. Microbiol. 252, 108924. doi: 10.1016/j.vetmic.2020.108924 33203576

[B32] García-Meniño I. García V. Mora A. Díaz-Jiménez D. Flament-Simon S. C. Alonso M. P.. (2018). Swine enteric colibacillosis in Spain: Pathogenic potential of mcr-1 ST10 and ST131 E. Coli Isolates. Front. Microbiol. 9. doi: 10.3389/fmicb.2018.02659 PMC6230658 30455680

[B33] García-Meniño I. Lumbreras P. Lestón L. Álvarez-Álvarez M. García V. Hammerl J. A.. (2022). Occurrence and genomic characterization of clone ST1193 clonotype 14-64 in uncomplicated urinary tract infections caused by escherichia coli in Spain. Microbiol. Spectr. 10, e00041-22. doi: 10.1128/spectrum.00041-22 35604206 PMC9241898

[B34] García-Meniño I. Lumbreras P. Valledor P. Díaz-Jiménez D. Lestón L. Fernández J.. (2020). Comprehensive Statistical Evaluation of Etest ^®^, UMIC ^®^, MicroScan and Disc Diffusion versus Standard Broth Microdilution: Workflow for an Accurate Detection of Colistin-Resistant and Mcr-Positive E. coli. Antibiotics 9 (12), 861. doi: 10.3390/antibiotics9120861 33287187 PMC7761637

[B35] Gupta K. Hooton T. M. Naber K. G. Wullt B. Colgan R. Miller L. G.. (2011). International clinical practice guidelines for the treatment of acute uncomplicated cystitis and pyelonephritis in women: A 2010 update by the Infectious Diseases Society of America and the European Society for Microbiology and Infectious Diseases. Clin Infect Dis. 52, e103-20–e120. doi: 10.1093/cid/ciq257 21292654

[B36] Ikuta K. S. Swetschinski L. R. Robles Aguilar G. Sharara F. Mestrovic T. Gray A. P.. (2022). Global mortality associated with 33 bacterial pathogens in 2019: a systematic analysis for the Global Burden of Disease Study 2019. Lancet 400, 2221–2248. doi: 10.1016/S0140-6736(22)02185-7 36423648 PMC9763654

[B37] Jaureguy F. Landraud L. Passet V. Diancourt L. Frapy E. Guigon G.. (2008). Phylogenetic and genomic diversity of human bacteremic Escherichia coli strains. BMC Genomics 9, 560. doi: 10.1186/1471-2164-9-560 19036134 PMC2639426

[B38] Joensen K. G. Scheutz F. Lund O. Hasman H. Kaas R. S. Nielsen E. M.. (2014). Real-time whole-genome sequencing for routine typing, surveillance, and outbreak detection of verotoxigenic escherichia coli. J. Clin. Microbiol. 52, 1501–1510. doi: 10.1128/JCM.03617-13 24574290 PMC3993690

[B39] Joensen K. G. Tetzschner A. M. M. Iguchi A. Aarestrup F. M. Scheutz F. (2015). Rapid and easy in silico serotyping of escherichia coli isolates by use of whole-genome sequencing data. J. Clin. Microbiol. 53, 2410–2426. doi: 10.1128/JCM.00008-15 25972421 PMC4508402

[B40] Johansson M. H. K. Bortolaia V. Tansirichaiya S. Aarestrup F. M. Roberts A. P. Petersen T. N. (2021). Detection of mobile genetic elements associated with antibiotic resistance in Salmonella enterica using a newly developed web tool: MobileElementFinder. J. Antimicrob. Chemother. 76, 101–109. doi: 10.1093/jac/dkaa390 33009809 PMC7729385

[B41] Johnson T. J. Danzeisen J. L. Youmans B. Case K. Llop K. Munoz-Aguayo J.. (2016). Separate F-type plasmids have shaped the evolution of the H30 subclone of escherichia coli sequence type 131. mSphere 1, 10.1128/msphere.00121-16. doi: 10.1128/mSphere.00121-16 PMC4933990 27390780

[B42] Johnson T. J. Elnekave E. Miller E. A. Munoz-Aguayo J. Figueroa C. F. Johnston B.. (2018). Phylogenomic analysis of extraintestinal pathogenic escherichia coli sequence type 1193, an emerging multidrug-resistant clonal group. Antimicrob. Agents Chemother. 63, 10.1128/aac.01913-18. doi: 10.1128/AAC.01913-18 PMC6325179 30348668

[B43] Kim Y. Oh T. Nam Y. S. Cho S. Y. Lee H. J. (2017). Prevalence of ST131 and ST1193 among bloodstream isolates of Escherichia coli not susceptible to ciprofloxacin in a tertiary care university hospital in Korea 2013-2014. Clin. Lab. 63, 1541–1543. doi: 10.7754/Clin.Lab.2017.170319 28879712

[B44] Klein R. D. Hultgren S. J. (2020). Urinary tract infections: microbial pathogenesis, host-pathogen interactions and new treatment strategies. Nat. Rev. Microbiol. 18, 211. doi: 10.1038/s41579-020-0324-0 32071440 PMC7942789

[B45] Klevens R. M. Edwards J. R. Richards C. L. Jr Horan T. C. Gaynes R. P. Pollock D. A.. (2007). Estimating health care-associated infections and deaths in U.S. hospitals 2002. Public Health Rep. 122, 160–166. doi: 10.1177/003335490712200205 17357358 PMC1820440

[B46] Kondratyeva K. Salmon-Divon M. Navon-Venezia S. (2020). Meta-analysis of pandemic escherichia coli ST131 plasmidome proves restricted plasmid-clade associations. Sci. Rep. 10, 1–11. doi: 10.1038/s41598-019-56763-7 31913346 PMC6949217

[B47] Kot B. (2019). Antibiotic resistance among uropathogenic escherichia coli. Polish J. Microbiol. 68, 403. doi: 10.33073/pjm-2019-048 PMC7260639 31880885

[B48] Kresken M. Körber-Irrgang B. Biedenbach D. J. Batista N. Besard V. Cantón R.. (2016). Comparative in *vitro* activity of oral antimicrobial agents against Enterobacteriaceae from patients with community-acquired urinary tract infections in three European countries. Clin. Microbiol. Infect. 22, 63.e1–63.e5. doi: 10.1016/j.cmi.2015.08.019 26321667

[B49] Lin W. H. Zhang Y. Z. Liu P. Y. Chen P. S. Wang S. N. Kuo P. Y.. (2021). Distinct Characteristics of Escherichia coli Isolated from Patients with Urinary Tract Infections in a Medical Center at a Ten-Year Interval. Pathog. (Basel Switzerland) 10, 1156. doi: 10.3390/pathogens10091156 PMC8469484 34578189

[B50] MacVane S. H. Tuttle L. O. Nicolau D. P. (2015). Demography and burden of care associated with patients readmitted for urinary tract infection. J. Microbiol. Immunol. Infect. 48, 517–524. doi: 10.1016/j.jmii.2014.04.002 24863498

[B51] Magiorakos A.-P. Srinivasan A. Carey R. B. Carmeli Y. Falagas M. E. Giske C. G.. (2012). Multidrug-resistant, extensively drug-resistant and pandrug-resistant bacteria: an international expert proposal for interim standard definitions for acquired resistance. Clin. Microbiol. Infect. 18, 268–281. doi: 10.1111/j.1469-0691.2011.03570.x 21793988

[B52] Mahérault A. C. Kemble H. Magnan M. Gachet B. Roche D. Nagard H.. (2019). Advantage of the F2:A1:B- IncF Pandemic Plasmid over IncC Plasmids in *In Vitro* Acquisition and Evolution of blaCTX-M Gene-Bearing Plasmids in Escherichia coli. Antimicrob. Agents Chemother. 63, 10.1128/aac.01130-19. doi: 10.1128/AAC.01130-19 PMC6761558 31332067

[B53] Mamani R. Flament-Simon S. C. García V. Mora A. Alonso M. P. López C.. (2019). Sequence types, clonotypes, serotypes, and virotypes of extended-spectrum β-lactamase-producing escherichia coli causing bacteraemia in a spanish hospital over a 12-year period, (2000 to 2011). Front. Microbiol. 10. doi: 10.3389/fmicb.2019.01530 PMC6646471 31379759

[B54] Manges A. R. Geum H. M. Guo A. Edens T. J. Fibke C. D. Pitout J. D. D. (2019). Global extraintestinal pathogenic escherichia coli (Expec) lineages. Clin. Microbiol. Rev. 32, e00135-18. doi: 10.1128/CMR.00135-18 31189557 PMC6589867

[B55] Medina M. Castillo-Pino E. (2019). An introduction to the epidemiology and burden of urinary tractinfections. Ther. Adv. Urol. 11, 1756287219832172. doi: 10.1177/1756287219832172 31105774 PMC6502976

[B56] Merino I. Shaw E. Horcajada J. P. Cercenado E. Mirelis B. Pallarés M. A.. (2016). CTX-M-15-H30Rx-ST131 subclone is one of the main causes of healthcare-associated ESBL-producing Escherichia coli bacteraemia of urinary origin in Spain. J. Antimicrob. Chemother. 71, 2125–2130. doi: 10.1093/jac/dkw133 27494832

[B57] Murray C. J. Ikuta K. S. Sharara F. Swetschinski L. Robles Aguilar G. Gray A.. (2022). Global burden of bacterial antimicrobial resistance in 2019: a systematic analysis. Lancet 399, 629–655. doi: 10.1016/S0140-6736(21)02724-0 35065702 PMC8841637

[B58] Nguyen Q. Nguyen T. T. N. Pham P. Chau V. Nguyen L. P. H. Nguyen T. D.. (2021). Genomic insights into the circulation of pandemic fluoroquinolone-resistant extra-intestinal pathogenic Escherichia coli ST1193 in Vietnam. Microb. Genomics 7 (12), 000733. doi: 10.1099/mgen.0.000733 PMC8767341 34904942

[B59] Nicolas-Chanoine M. H. Bertrand X. Madec J. Y. (2014). Escherichia coli ST131, an intriguing clonal group. Clin. Microbiol. Rev. 27, 543–574. doi: 10.1128/CMR.00125-13 24982321 PMC4135899

[B60] Peirano G. Bradford P. A. Kazmierczak K. M. Badal R. E. Hackel M. Hoban D. J.. (2014). Global incidence of carbapenemase-producing escherichia coli ST131. Emerg. Infect. Dis. 20, 1928. doi: 10.3201/eid2011.141388 25340464 PMC4214325

[B61] Peirano G. Pitout J. D. D. (2019). Extended-spectrum β-lactamase-producing enterobacteriaceae: update on molecular epidemiology and treatment options. Drugs 79, 1529–1541. doi: 10.1007/s40265-019-01180-3 31407238

[B62] Pitout J. D. DeVinney R. (2017). Escherichia coli ST131: a multidrug-resistant clone primed for global domination. F1000Res 6, F1000 Faculty Rev-195. doi: 10.12688/f1000research.10609.1 PMC5333602 28344773

[B63] Pitout J. D. D. Peirano G. Chen L. DeVinney R. Matsumura Y. (2022). Escherichia coli ST1193: Following in the Footsteps of E. coli ST131. Antimicrob. Agents Chemother 66, e00511-22. doi: 10.1128/AAC.00511-22 35658504 PMC9295538

[B64] Poirel L. Rodriguez-Martinez J. M. Mammeri H. Liard A. Nordmann P. (2005). Origin of plasmid-mediated quinolone resistance determinant QnrA. Antimicrob. Agents Chemother. 49, 3523–3525. doi: 10.1128/AAC.49.8.3523-3525.2005 16048974 PMC1196254

[B65] Price L. B. Johnson J. R. Aziz M. Clabots C. Johnston B. Tchesnokova V.. (2013). The epidemic of extended-spectrum-β-lactamase-producing Escherichia coli ST131 is driven by a single highly pathogenic subclone, H30-Rx. MBio 4. doi: 10.1128/mBio.00377-13 PMC3870262 24345742

[B66] Roer L. Johannesen T. B. Hansen F. Stegger M. Tchesnokova V. Sokurenko E.. (2018). CHTyper, a Web Tool for Subtyping of Extraintestinal Pathogenic Escherichia coli Based on the fumC and fimH Alleles. J. Clin. Microbiol. 56, e00063–e00018. doi: 10.1128/JCM.00063-18 29436420 PMC5869820

[B67] Spurbeck R. R. Dinh P. C. Walk S. T. Stapleton A. E. Hooton T. M. Nolan L. K.. (2012). Escherichia coli Isolates That Carry vat, fyuA, chuA, and yfcV Efficiently Colonize the Urinary Tract. Infect. Immun. 80, 4115–4122. doi: 10.1128/IAI.00752-12 22966046 PMC3497434

[B68] Taati Moghadam M. Mirzaei M. Fazel Tehrani Moghaddam M. Babakhani S. Yeganeh O. Asgharzadeh S.. (2021). The challenge of global emergence of novel colistin-resistant escherichia coli ST131. Microb Drug Resist. 27(11), 1513–1524. doi: 10.1089/MDR.2020.0505 33913748

[B69] Talan D. A. Takhar S. S. Krishnadasan A. Abrahamian F. M. Mower W. R. Moran G. J. (2016). Fluoroquinolone-resistant and extended-spectrum β-lactamase–producing escherichia coli infections in patients with pyelonephritis, United States. Emerg. Infect. Dis. 22, 1594. doi: 10.3201/eid2209.160148 27532362 PMC4994338

[B70] Tchesnokova V. Larson L. Basova I. Sledneva Y. Choudhury D. Heng J.. (2023). Increase in the Rate of Gut Carriage of Fluoroquinolone-Resistant Escherichia coli despite a Reduction in Antibiotic Prescriptions. Res. Sq 3, 110. doi: 10.21203/rs.3.rs-2426668/v1 PMC10421857 37567971

[B71] Tchesnokova V. L. Rechkina E. Larson L. Ferrier K. Weaver J. L. Schroeder D. W.. (2019). Rapid and extensive expansion in the United States of a new multidrug-resistant escherichia coli clonal group, sequence type 1193. Clin. Infect. Dis. 68, 334–337. doi: 10.1093/cid/ciy525 29961843 PMC6321845

[B72] Tetzschner A. M. M. Johnson J. R. Johnston B. D. Lund O. Scheutz F. (2020). In silico genotyping of escherichia coli isolates for extraintestinal virulence genes by use of whole-genome sequencing data. J. Clin. Microbiol. 58, 10.1128/jcm.01269-20. doi: 10.1128/JCM.01269-20 PMC7512150 32669379

[B73] Valenza G. Werner M. Eisenberger D. Nickel S. Lehner-Reindl V. Höller C.. (2019). First report of the new emerging global clone ST1193 among clinical isolates of extended-spectrum β-lactamase (ESBL)-producing Escherichia coli from Germany. J. Glob. Antimicrob. Resist. 17, 305–308. doi: 10.1016/j.jgar.2019.01.014 30682563

[B74] Weissman S. J. Johnson J. R. Tchesnokova V. Billig M. Dykhuizen D. Riddell K.. (2012). High-resolution two-locus clonal typing of extraintestinal pathogenic escherichia coli. Appl. Environ. Microbiol. 78, 1353–1360. doi: 10.1128/AEM.06663-11 22226951 PMC3294456

[B75] WHO (2014). Antimicrobial resistance: global report on surveillance WHO/HSE/PED/AIP/2014.2. (Geneva, Switzerland: WHO Document Production Services).

[B76] Wick R. R. Judd L. M. Gorrie C. L. Holt K. E. (2017). Unicycler: Resolving bacterial genome assemblies from short and long sequencing reads. PloS Comput. Biol. 13, e1005595. doi: 10.1371/journal.pcbi.1005595 28594827 PMC5481147

[B77] Wirth T. Falush D. Lan R. Colles F. Mensa P. Wieler L. H.. (2006). Sex and virulence in Escherichia coli : an evolutionary perspective. Mol. Microbiol. 60, 1136–1151. doi: 10.1111/j.1365-2958.2006.05172.x 16689791 PMC1557465

[B78] Yamaji R. Friedman C. R. Rubin J. Suh J. Thys E. McDermott P.. (2018a). A population-based surveillance study of shared genotypes of escherichia coli isolates from retail meat and suspected cases of urinary tract infections. mSphere 3 (4), e00179-18. doi: 10.1128/mSphere.00179-18 30111626 PMC6094058

[B79] Yamaji R. Rubin J. Thys E. Friedman C. R. Riley L. W. (2018b). Persistent pandemic lineages of uropathogenic escherichia coli in a college community from 1999 to 2017. J. Clin. Microbiol. 56 (4), e01834-17. doi: 10.1128/JCM.01834-17 29436416 PMC5869836

[B80] Zankari E. Allesøe R. Joensen K. G. Cavaco L. M. Lund O. Aarestrup F. M. (2017). PointFinder: a novel web tool for WGS-based detection of antimicrobial resistance associated with chromosomal point mutations in bacterial pathogens. J. Antimicrob. Chemother. 72, 2764–2768. doi: 10.1093/jac/dkx217 29091202 PMC5890747

