# Supplementary material for: Fluoroquinolone resistance in complicated urinary tract infections: association with the increased occurrence and diversity of Escherichia coli of clonal complex 131, together with ST1193
Source: Front Cell Infect Microbiol. 2024 Feb 27;14:1351618. doi: 10.3389/fcimb.2024.1351618 (PMC10953827; doi:10.3389/fcimb.2024.1351618)
Supplement: Supplementary file 1 [file DataSheet_1.docx]

Supplementary Material

**Fluoroquinolone resistance in complicated urinary tract infections: association with the increased occurrence and diversity of Escherichia coli of Clonal Complex 131, together with ST1193**

Isidro García-Meniño, Vanesa García, Pilar Lumbreras-Iglesias, Javier Fernández, Azucena Mora

**Correspondence:** [azucena.mora@usc.es](mailto:azucena.mora@usc.es)

**PCR reactions**

Briefly, overnight-grown onto Tryptone Soy agar was picked with a 1 µl inoculation loop and suspended in 600 µl of sterile Milli-Q water. Bacterial suspensions were boiled at 100 °C for 5 min and then centrifuged for 2 min at 11,000 rpm to pellet bacterial debris. The supernatant was used as DNA template in PCR.

All PCR reactions were done in a final reaction volume of 25 µl, using 5 µl of DNA template, 12.5 µl of NZYTaq 2x Green MasterMix, 0.2-0.5 µM of each primer, and up to 25 µl of sterile Milli-Q water. PCR amplifications were performed on Applied Biosystem 2720 Thermal Cycler using an initial heat activation step of 3 min at 94 °C; then 35 cycles of 1 min at 94 °C, 1 min at specific annealing temperature of the primer set used, and 90 min at 72 °C; and a final extension step of 3 min at 72 °C.

PCR products were separated through 1.5% agarose gel (Seakem LE agarose, Lonza) containing Green Safe Premium (Nzytech) (3 µl/100 ml agarose) by convectional electrophoresis and the amplified PCR products were visualized using Gel Doc XR (BioRad, CA).

The primers, the amplicon size and the specific annealing T for each PCR reaction are indicated in the Supplementary Tables 4 to 9.

**Table S2.** Targets and primers used to determine the UPEC status

| **Target** | **Primers** | **Nucleotide sequence (5´- 3´)** | **Size (bp)** | **Annealing T (°C)** | **Reference** |
| --- | --- | --- | --- | --- | --- |
| *vat* | vat-F | TCAGGACACGTTCAGGCATTCAGT | 1100 | 70 | (Spurbeck et al., 2012) |
|  | vat-R | GGCCAGAACATTTGCTCCCTTGTT |  |  |  |
| *chu*A | ChuA-F | CTGAAACCATGACCGTTACG | 652 | 63 |  |
|  | ChuA-R | TTGTAGTAACGCACTAAACC |  |  |  |
| *fyu*A | fyuA-F | GTAAACAATCTTCCCGCTCGGCAT | 850 | 63 |  |
|  | fyuA-R | TGACGATTAACGAACCGGAAGGGA |  |  |  |
| *yfc*V | yfcV-F | ACATGGAGACCACGTTCACC | 292 | 63 |  |
|  | YfcV-R | GTAATCTGGAATGTGGTCAGG |  |  |  |

**Table S3.** Primers used for the *rbf*O25, H4 (*fliC_H4_*) and H5 (*fliC_H5_*) screening

| **Target** | **Primers** | **Nucleotide sequence (5´- 3´)** | **Size (bp)** | **Annealing T (°C)** | **Reference** |
| --- | --- | --- | --- | --- | --- |
| *rfb*O25b | rfbO25b.r | TGCTATTCATTATGCGCAGC | 300 | 56 | (Clermont et al., 2008) |
|  | rfb.1bis | ATACCGACGACGCCGATCTG |  |  |  |
| *fli*C_H4_ | H4-F | GCAGCGTATTCGTGAACTGA | 713 | 66 | (Mora et al., 2011) |
|  | H4-R | GCTGGATAATCTGCGCTTTC |  |  |  |
| *fli*C_H5_ | H5-F2 | Ggatgaaattgatcgcgttt | 477 | 48-52 | This study (GenBank accession references AY249990 and AY337469) |
|  | H5-R | GTAGCCGCAGTCGTTAGTCC |  |  |  |

**Table S4.** Primers used for the detection and/or sequencing of *bla* and *mcr* genes

| **Target** | **Primers** | **Nucleotide sequence (5´- 3´)** | **Size (bp)** | **Annealing T (°C)** | **Reference** |
| --- | --- | --- | --- | --- | --- |
| *bla*_CTX-M_ | CTX-C3 | ATGTGCAGCACCAGTAAAGTGATG | 542 | 55 | (Mora et al., 2013) |
|  | CTX-C4 | ACCGCGATATCGTTGGTGGTGCC |  |  |  |
| *bla*_CTX-M_ group1 | M13U | GGTTAAAAAATCACTGCGTC | 863 | 60 | (Saladin et al., 2002) |
|  | M13L | TTGGTGACGATTTTAGCCGC |  |  |  |
| *blaCTX‐M-*group1 | ^a^CTX-15-F1 | GAAGCTAATAAAAAACACACGTGG | 1044-1123 | 52 | (Mora et al., 2013) |
|  | ^a^CTX-15-R | GTATGCGCAAGCGCAGGTGG |  |  |  |
| *bla*_CTX-M_ group9 | CTX-M9-F | GTGACAAAGAGAGTGCAACGG | 856 | 64 | (Simarro et al., 2000) |
|  | CTX-M9-R | ATGATTCTCGCCGCTGAAGCC |  |  |  |
| *bla*_CTX-M_ group9 | ^a^CTX-M9-14-14B-24F | GAATACTGATGTAACACGGA | 998 | 44 | (García-Meniño et al., 2018) |
|  | ^a^CTX-M9-R | AGCTGAAGATGTATATCAAG |  |  |  |
| *bla*_CTX-M_ group9 | ^a^CTX-M9-14-14B-24F | GAATACTGATGTAACACGGA | 989 | 52 | (García-Meniño et al., 2018) |
|  | ^a^CTX-M14-24-R | CTGCGTTGTCGGGAAGATACG |  |  |  |
| *bla*_CTX-M_ group9 | ^a^CTX-M9-14B-F | CCTATACCCGAGGCGCGACAG | 1059 | 44 | (García-Meniño et al., 2018) |
|  | ^a^CTX-M9-R | AGCTGAAGATGTATATCAAG |  |  |  |
| *bla*_CTX-M_ group9 | ^a^CTX-M14-24-F | CTAAATTCTTCGTGAAATAGTG | 1049 | 44 | (García-Meniño et al., 2018) |
|  | ^a^CTX-M14-24-R | CTGCGTTGTCGGGAAGATACG |  |  |  |
| *bla*_SHV_ | SHV-F2 | TTGTCGCTTCTTTACTCGCC | 879 | 64 | (Mora et al., 2013) |
|  | SHV-R2 | CCCGGCGATTTGCTGATTTCGC |  |  |  |
| *LAT-1 to LAT-4,*  *CMY-2 to CMY-7, BIL-1* | CITMF | TGGCCAGAACTGACAGGCAAA | 462 | 66 | (Pérez-Pérez and Hanson, 2002) |
|  | CITMR | TTTCTCCTGAACGTGGCTGGC |  |  |  |
| *mcr-1* | mcr1_320bp_fw | AGTCCGTTTGTTCTTGTGGC | 320 | 58 | (Rebelo et al., 2018) |
|  | mcr1_320bp_rev | AGATCCTTGGTCTCGGCTTG |  |  |  |
| *mcr-2* | mcr2_700bp_fw | CAAGTGTGTTGGTCGCAGTT | 715 | 58 | (Rebelo et al., 2018) |
|  | mcr2_700bp_rev | TCTAGCCCGACAAGCATACC |  |  |  |
| *mcr-3* | mcr3_900bp_fw | AAATAAAAATTGTTCCGCTTATG | 929 | 58 | (Rebelo et al., 2018) |
|  | mcr3_900bp_rev | AATGGAGATCCCCGTTTTT |  |  |  |
| *mcr-4* | mcr4_1100bp_fw | TCACTTTCATCACTGCGTTG | 1116 | 58 | (Rebelo et al., 2018) |
|  | mcr4_1100bp_rev | TTGGTCCATGACTACCAATG |  |  |  |
| *mcr-5* | MCR5_FW | ATGCGGTTGTCTGCATTTATC | 1644 | 50 | (Borowiak et al., 2017) |
|  | MCR5_RV | TCATTGTGGTTGTCCTTTTCTG |  |  |  |

**Table S5**. Targets and primers used for the phylogroup determination in *E. coli*

| **Target** | **Primers** | **Nucleotide sequence (5´- 3´)** | **Size (bp)** | **Annealing Tª (°C)** | **Reference** |
| --- | --- | --- | --- | --- | --- |
| *chuA* | chuA.1b | ATGGTACCGGACGAACCAAC | 288 | 58 | (Clermont et al., 2013) |
|  | chuA.2 | TGCCGCCAGTACCAAAGACA |  |  | (Clermont et al., 2000) |
| *yjaA* | yjaA.1b | CAAACGTGAAGTGTCAGGAG | 211 |  | (Clermont et al., 2013) |
|  | yjaA.2b | AATGCGTTCCTCAACCTGTG |  |  |  |
| *TspE4C2* | TspE4C2.1b | CACTATTCGTAAGGTCATCC | 152 |  | (Clermont et al., 2013) |
|  | TspE4C2.2b | AGTTTATCGCTGCGGGTCGC |  |  |  |
| *arpA* | AceK.f | AACGCTATTCGCCAGCTTGC | 400 |  | (Clermont et al., 2013) |
|  | ArpA1.r | TCTCCCCATACCGTACGCTA |  |  |  |
| *trpAgpC (C)* | trpAgpC.1 | AGTTTTATGCCCAGTGCGAG | 219 | 56 | (Lescat et al., 2013) |
|  | trpAgpC.2 | TCTGCGCCGGTCACGCCC |  |  |  |
| *arpA (E)* | ArpAgpE.f | GATTCCATCTTGTCAAAATATGCC | 301 | 57 | (Lescat et al., 2013) |
|  | ArpAgpE.r | GAAAAGAAAAAGAATTCCCAAGAG |  |  |  |
| *trpA* | trpBA.f | CGGCGATAAAGACATCTTCAC | 489 | 56 | (Clermont et al., 2008) |
|  | trpBA.r | GCAACGCGGCCTGGCGGAAG |  |  |  |
| *ybgD (G)* | *ybgD.1* | TATGCGGCTGATGAAGGATC | 177 | 59 | (Clermont et al., 2019) |
|  | *ybgD.2* | GTTGACTAAGCGCAGGTCGA |  |  |  |
| *cfaB (F)* | *cfaB.1* | CTAACGTTGATGCTGCTCTG | 384 |  | (Clermont et al., 2019) |
|  | *cfaB.2* | TGCTAACTACGCCACGGTAG |  |  |  |

**Table S6**. Targets and primers to determine clonotypes (CH) and sequence types (ST)

| **Target** | **Primers** | **Nucleotide sequence (5´- 3´)** | **Locus size (bp)** | **Reference** |
| --- | --- | --- | --- | --- |
| *fimH* | fimH-F | CACTCAGGGAACCATTCAGGCA | 489 | (Weissman et al., 2012) |
|  | fimH-R | CTTATTGATAAACAAAAGTCAC |  |  |
| *adk* | adkF | ATTCTGCTTGGCGCTCCGGG | 536 | (Wirth et al., 2006) |
|  | adkR | CCGTCAACTTTCGCGTATTT |  |  |
| *fumC* | fumCF | TCACAGGTCGCCAGCGCTTC | 469 |  |
|  | fumCR | GTACGCAGCGAAAAAGATTC |  |  |
| *gyrB* | gyrBF | TCGGCGACACGGATGACGGC | 460 |  |
|  | gyrBR | ATCAGGCCTTCACGCGCATC |  |  |
| *icd* | icdF | ATGGAAAGTAAAGTAGTTGTTCCGGCACA | 518 |  |
|  | icdR | GGACGCAGCAGGATCTGTT |  |  |
| *mdh* | mdhF | ATGAAAGTCGCAGTCCTCGGCGCTGCTGGCGG | 452 |  |
|  | mdhR | TTAACGAACTCCTGCCCCAGAGCGATATCTTTCTT |  |  |
| *purA* | purAF | CGCGCTGATGAAAGAGATGA | 478 |  |
|  | purAR | CATACGGTAAGCCACGCAGA |  |  |
| *recA* | recAR1 | AGCGTGAAGGTAAAACCTGTG | 510 |  |
|  | recAF1 | ACCTTTGTAGCTGTACCACG |  |  |

Allele assignments for *fimH* were determined using the fimtyper database available at the Center for Genomic Epidemiology (CGE) website <https://bitbucket.org/genomicepidemiology/fimtyper_db/downloads>, and the two-locus approach—(*fumC*/*fimH*) typing determined the CH “type”. the assigned of STs was conducted through the EnteroBase website (<http://mlst.warwick.ac.uk/mlst/dbs/Ecoli>).

**Table S7.** Targets and primers used in the virotype scheme of CC131 isolates

| **Target** | **Primers** | **Nucleotide sequence (5´- 3´)** | **Size (bp)** | **Annealing Tª (°C)** | **Reference** |
| --- | --- | --- | --- | --- | --- |
| *afa/draBC* | afa1 | GCTGGGCAGCAAACTGATAACTCTC | 750 | 64 | (Le Bouguenec et al., 1992) |
|  | afa2 | CATCAAGCTGTTTGTTCGTCCGCCG |  |  |  |
| *afaFM955459* | Afa-025F | GAGTCACGGCAGTCGCGGCGG | 207 | 55 | (Blanco et al., 2009) |
|  | Afa-025R | TTCACCGGCGACCAGCCATCTCC |  |  |  |
| *iroN* | Ironec-f | AAGTCAAAGCAGGGGTTGCCCG | 665 | 62 | (Johnson et al., 2000) |
|  | Ironec-r | GACGCCGACATTAAGACGCAG |  |  |  |
| *sat* | SatF | GCAGCTACCGCAATAGGAGGT | 937 | 60 | (Johnson et al., 2003) |
|  | SatR | CATTCAGAGTACCGGGGCCTA |  |  |  |
| *ibeA* | Ibe10 f | AGGCAGGTGTGCGCCGCGTAC | 170 | 58 | (Johnson and Stell, 2000) |
|  | Ibe10 r | TGGTGCTCCGGCAAACCATGC |  |  |  |
| *papGII* | Pap-II f | GGGCATTGCTACGGTAACCTG | 545 | 54-60 | (Mora et al., 2013) |
|  | Pap-II r | CGCTATTAATAGACAGATCACC |  |  |  |
| *papGIII* | Pap-III f | CGGCAACTTTAAGCTATGTG | 720 | 60-68 | (Mora et al., 2013) |
|  | Pap-III r | TGTACCATCTCATCGTTGTCTC |  |  |  |
| *cnf1* | CNF1-F2 | CAGGAGGTACTTAGCAGCGT | 468 | 48-58 | (Mora et al., 2013) |
|  | CNF1-RC | TAATTTTGGGTTTGTATC |  |  |  |
| *hlyA* | hly f | AACAAGGATAAGCACTGTTCTGGCT | 1177 | 64 | (Yamamoto et al., 1995) |
|  | hly r | ACCATATAAGCGGTCATTCCCGTCA |  |  |  |
| *cdtB* | cdt-s1 | GAAAGTAAATGGAATATAAATGTCCG | 466 | 48-52 | (Tóth et al., 2003) |
|  | cdt-as1 | AAATCTCCTGCAATCATCCAGTTA |  |  |  |
|  | cdt-s2 | GAAAATAAATGGAACACACATGTCCG |  |  |  |
|  | cdt-as2 | AAATCACCAAGAATCATCCAGTTA |  |  |  |
| *neuC-K1* | neu1 | AGGTGAAAAGCCTGGTAGTGTG | 676 | 54 | (Moulin-Schouleur et al., 2006) |
|  | neu2 | GGTGGTACATCCCGGGATGTC |  |  |  |
| *Kps-M II-K2* | kpsII f | GCGCATTTGCTGATACTGTTG | 570 | 60 | (Johnson and O’Bryan, 2004) |
|  | KpsII-K2r | AGGTAGTTCAGACTCACACCT |  |  |  |
| *Kps-M II-K5* | K5 f | CAGTATCAGCAATCGTTCTGTA | 159 | 54 | (Johnson and Stell, 2000) |
|  | kpsII r | CATCCAGACGATAAGCATGAGCA |  |  |  |

**Table S8.** Virotype designation scheme for CC131 *E. coli* (adapted from Dahbi et al., 2014).

| **VIROTYPES** | *afa/draBC* | *afa operon FM955459* | *iroN* | *sat* | *ibeA* | *papG II* | *papG III* | *cnf1* | *hlyA* | *cdtB* | *neuCK1* | *kpsM II-K2* | *kpsM II-K5* |
| --- | --- | --- | --- | --- | --- | --- | --- | --- | --- | --- | --- | --- | --- |
| **Virotype A** | + | + | - | +/- | - | - | - | - | - | - | - | + | - |
| **Virotype B** | - | - | + | +/- | - | +/- | - | - | - | - | - | - | +/- |
| **Virotype C1** | - | - | - | + | - | - | - | - | - | - | - | + | - |
| **Virotype C2** | - | - | - | + | - | - | - | - | - | - | - | - | + |
| **Virotype C3** | - | - | - | + | - | - | - | - | - | - | - | - | - |
| **Virotype D1** | - | - | +/- | - | + | - | - | - | - | + | - | - | + |
| **Virotype D2** | - | - | +/- | - | + | - | + | - | - | + | - | - | + |
| **Virotype D3** | +/- | +/- | +/- | +/- | + | - | - | - | - | - | - | - | + |
| **Virotype D4** | - | - | +/- | - | + | - | - | - | - | - | + | - | - |
| **Virotype D5** | - | - | +/- | - | + | - | + | + | + | - | - | - | + |
| **Virotype E** | - | - | - | - | - | + | - | + | + | - | - | - | + |
| **Virotype F** | - | - | - | - | - | + | - | - | - | - | - | - | + |

**Table S9.** *In silico* characterization and phenotypic AMR of the cUTI ESBL-producing isolates belonging to the CC131

| **^1^ID code genome; isolate** | **^2^O:H antigens** | **^3^ST#1**  **/ST#2** | **^4^cgST** | **^5^CHType** | **^6^Acquired resistances and point mutations (in bold)** | **^7^Plasmid content**  **Inc. group [pMLST]** | **^8^Virulence genes** | **^9^Mobile genetic elements (& relation to AMR and virulence traits)** | **^10^CRISPR-Cas system /**  **Cas-type/subtype** | **^11^Phenotypic AMR** |
| --- | --- | --- | --- | --- | --- | --- | --- | --- | --- | --- |
| **LREC-278**  FVL7/22 | O25:H4 | 131/43 | 12614 | 40-30 | *bla*_CTX-M-15_**,** *aadA5*, *mdf(A)*, *mph(A)*, *sul1*, *tet(A)*, *dfrA17*, *sitABCD*, *qacE*  ***gyrA* p.S83L, *gyrA* p.D87N, *parC* p.S80I, *parC* p.E84V, *parE* p.I529L** | IncF [F31:A4:B1]  ColMG828-like | *anr, csgA, chuA, fdeC; fimH; fyuA, gad, hra, iha, irp2, iss, iucC, iutA, kpsE, kpsMII K5, nlpl; ompT, papA F43, papC, sat, senB, shiB; sitA, terC, traJ; traT, usp, yehA, yehB, yehC, yehD, yfcV* | IS30 (*iha*), IS6100 (*aadA5, mph(A), qacE, dfrA17, sul1*), ISEc43 (*papA F43*, *papC*) , ISEc53 (*fimH*), Tn5403 (*tet(A)*), MITEEc1 (*terC*), MITEEc1 (*yehA*, *yehB*, *yehC*, *yehD*) | - | AMP-AM, PIP, TIC, AMC, CXM, CIX, CTX, CAZ, FEP, AZT, NOR, CIP, LEV, TOB, AMI, TMP, TMP-SXT |
| **LREC-279**  FVL25/22 | O25:H4 | 9126/43 | 7829 | 1267-30 | *bla*_CTX-M-15_**,** *mdf(A)*, *sitABCD*  ***gyrA* p.S83L, *gyrA* p.D87N, *parC* p.S80I, *parC* p.E84V, *parE* p.I529L** | IncF [F40*:A-:B34*]  ColMG828-like  Col156-like | *chuA, colE2-like, csgA, faeC, faeD, faeF, faeH, faeI, fdeC, fimH, fyuA, gad, iha, irp2, iss, iucC, iutA, kpsE, kpsMII K5, nlpl, ompT, papA F43, sat, sitA, terC, usp, yehA, yehB, yehC, yehD, yfcV* | ISEc9 (*bla*_CTX-M-15_, *yfcV*), IS629 (*iutA, papA F43, iucC, sat*), IS640 (*faeC, faeD, faeF, faeH, faeI),* ISEc38 (*fyuA, irp2*), ISEc53 (*fimH*), MITEEc1 (*terC*), MITEEc1 (*yehA*, *yehB*, *yehC*, *yehD*) | CAS-TYPE_U  Cas2_0_I-II-III | AMP-AM, PIP, TIC, CXM, CIX, CTX, CAZ, FEP, AZT, NOR, CIP, LEV |
| **LREC-280**  FVL28/22 | O25:H4 | 131/43 | 142625 | 40-30 | *bla*_CTX-M-15_, *bla*_TEM-1A_, *aadA2*, *mdf(A)*, *mph(A)*, *sul1*, *dfrA12*, *sitABCD*, *qacE*  ***gyrA* p.S83L, *gyrA* p.D87N, *parC* p.S80I, *parC* p.E84V, *parE* p.I529L** | IncF [F4:A1:B20]  IncB/O/K/Z  ColBS512-like  ColMG828-like  ColMP18-like | *anr, chuA, cnf1, csgA, fdeC, fimH, fyuA, gad, hlyA, hra, iha, irp2, iss, iucC, iutA, kpsE, kpsMII K5, nlpl, ompT, papA F43, papC, sat, shiB, sitA, terC, traT, usp, yehA, yehB, yehC, yehD, yfcV* | ISEc9 (*bla*_CTX-M-15,_ *fdeC*), IS30 (*iutA, iucC, sat*), IS6100 (*aadA2, mph(A), dfrA12, sul1, qacE*), IS629 (*iutA, iucC, sat*), IS682 (*cnf1*), ISEc53 (*fimH*), ISKpn37 (*hlyA*), Tn801 (*bla*_TEM-1A_), MITEEc1 (*terC*), MITEEc1 (*yfcV, yehA, yehB, yehC, yehD*) | - | AMP-AM, PIP, TIC, PIP-TAZ, AMC, CXM, CIX, CTX, CAZ, FEP, CTN-TAZ, AZT, NOR, CIP, LEV, TMP, TMP-SXT |
| **LREC-281**  FVL34/22 | O25:H4 | 131/43 | 116708 | 40-30 | *bla*_CTX-M-15_**,** *bla*_OXA-1_, *aac(3)-IIa*, *aac(6’)-Ib-cr*, *qnrS1,* *mdf(A)*, *dfrA14, catB3*, *sitABCD*  ***gyrA* p.S83L, *gyrA* p.D87N, *parC* p.S80I, *parC* p.E84V, *parE* p.I529L** | IncF [F4:A-:B10]  IncN (ST6) | *chuA, cnf1, csgA, fdeC, fimH, fyuA, gad, hlyA, hra, iha, irp2, iss, iucC, iutA, kpsE, kpsMII K5, ompT, papA F43, papC, sat, senB, shiB, sitA, terC, traJ, traT, usp, yehA, yehB, yehC, yehD, yfcV* | IS30 (*sat, iucC, iutA*), IS6100 (*dfrA14*), ISKpn8 (*gad*), MITEEc1 (*terC*) | CAS-TypeI  Cas3_0_I | AMP-AM, PIP, TIC, AMC, CXM, CIX, CTX, CAZ, FEP, AZT, NOR, CIP, LEV, GEN, TOB, AMI, TMP |
| LREC-283  FVL59/22 | O25:H4 | 131/43 | 29126 | 40-30 | *bla*_CTX-M-15_, *bla*_TEM-1B_, *mdf(A)*, *sitABCD*  ***gyrA* p.S83L, *gyrA* p.D87N, *parC* p.S80I, *parC* p.E84V, *parE* p.I529L** | IncF [F-:A2:B-] | *chuA, cnf1, csgA, fdeC, fimH, fyuA, gad, hha, hra, irp2, iss, kpsMII K5, nlpl, ompT, papA F13, papC, sitA, terC, usp, yehA, yehB, yehC, yehD* | ISEc9 (*bla*_CTX-M-15_), IS5 (*bla*_TEM-1B_), IS26 (*bla*_TEM-1B_), IS682(*cnf1, papC, papA F13*), ISEc53 (*fimH*), MITEEc1 (*yehA, yehB, yehC, yehD),* MITEEc1 (*ompT*), MITEEc1 (*terC*) | - | AMP-AM, PIP, TIC, AMC, CXM, CIX, CTX, CAZ, FEP, AZT, NOR, CIP, LEV |
| LREC-284  FVL79/22 | O25:H4 | 131/43 | 29126 | 40-30 | *bla*_CTX-M-15_, *bla*_TEM-1B_, *mdf(A)*, *sitABCD*  ***gyrA* p.S83L, *gyrA* p.D87N, *parC* p.S80I, *parC* p.E84V, *parE* p.I529L** | IncF [F-:A2:B20] | *chuA, cnf1, csgA, fdeC, fimH, fyuA, gad, hlyA, hra, iha, irp2, iss, iucC, iutA, kpsE, nlpl, ompT, papA F13, papA F43, papC, sat, sitA, terC, usp, yehA, yehB, yehC, yehD, yfcV* | ISEc9 (*bla*_CTX-M-15_), IS26 (*bla*_TEM-1B_), ISKpn8 (*bla*_TEM-1B_), ISEc53 (*fimH*), MITEEc1 (*yehA, yehB, yehC, yehD, yfcV*), MITEEc1 (*terC*) | - | AMP-AM, PIP, TIC, AMC, CXM, CIX, CTX, CAZ, FEP, AZT, NOR, CIP, LEV |
| **LREC-285**  FVL93/22 | O25:H4 | 9126/43 | 7829 | 1267-30 | *bla*_CTX-M-15_**,** *bla*_TEM-1B_, *bla*_OXA-1_, *aac(6’)-Ib-cr, mdf(A)*, *catB3*, *sitABCD*  ***gyrA* p.S83L, *gyrA* p.D87N, *parC* p.S80I, *parC* p.E84V, *parE* p.I529L** | IncF [F-:A1:B-]  ColMG828-like  Col156-like | *chuA, colE2-like, csgA, fdeC, fimH, fyuA, gad, iha, irp2, iss, iucC, iutA, kpsE, kpsMII K5, nlpl, ompT, papA F43, sat, sitA, terC, traJ, traT, usp, yehA, yehB, yehC, yehD, yfcV* | ISEc9 (*bla*_CTX-M-15_, *bla*_TEM-1B_, *traJ*, *traT*), IS629 (*iutA, papA F43, sat, iucC*), ISEc53 (*fimH*), MITEEc1 (*yfcV*), MITEEc1 (*terC*) | - | AMP-AM, PIP, TIC, AMC, CXM, CIX, CTX, CAZ, FEP, AZT, NOR, CIP, LEV, TOB, AMI |
| **LREC-286**  FVL96/22 | O25:H4 | 131/43 | 139233 | 40-30 | *bla*_CTX-M-15_**,** *bla*_OXA-1_, *aadA5*, *aac(6’)-Ib-cr*, *mdf(A)*, *mph(A)*, *catB3*, *sul1*, *dfrA17*, *sitABCD,* *qacE*  ***gyrA* p.S83L, *gyrA* p.D87N, *parC* p.S80I, *parC* p.E84V, *parE* p.I529L** | IncF [F2:A1:B-] | *afaA, afaC, afaD, chuA, csgA, fdeC, fimH, fyuA, gad, iha, irp2, iss, iucC, iutA, kpsE, kpsMII K5,* *nfaE, nlpl, ompT, papA F43, sat, shiA, sitA, terC, usp, yehB, yehC, yehD, yfcV* | IS6100 (*mph(A), sul1, qacE*), ISEc10 (*kpsE*), ISEc38 (*irp2, fyuA*), MITEEc1 (*ompT*), MITEEc1 (*terC*), MITEEc1 (*yfcV*) | CAS-TypeI  Cas3_0_I | AMP-AM, PIP, TIC, AMC, CXM, NOR, CIP, LEV, TOB, TMP, TMP-SXT |
| **LREC-287**  FVL103/22 | O25:H4 | 131/43 | 122338 | 40-30 | *bla*_CTX-M-15_**,** *bla*_OXA-1_, *bla*_TEM-1B_, *aadA5*, *aph(6)-Id*, *aph(3’’)-Ib*, *aac(6’)-Ib-cr*, *mdf(A)*, *mph(A)*, *sul1*, *sul2*, *tet(A)*, *dfrA17*, *sitABCD,* *qacE*  ***gyrA* p.S83L, *gyrA* p.D87N, *parC* p.S80I, *parC* p.E84V, *parE* p.I529L** | IncF [F29:A-:B10]  ColMG828-like  Col440I-like | *chuA, csgA, fdeC, fimH, fyuA, gad, iha, irp2, iss, iucC, iutA, kpsE, kpsMII K5, nlpl, ompT, papA F43, sat, senB, shiB, sitA, terC, traJ, traT, usp, yehA, yehB, yehC, yehD, yfcV* | ISEc9 (*bla*_CTX-M-15_), IS6100 (*aadA5, mph(A), dfrA17, sul1, qacE*), , ISEc38 (*fyuA, irp2*), MITEEc1 (*iss, csgA*), MITEEc1 (*yfcV, yehA, yehB, yehC, yehD*), MITEEc1 (*terC*) | CAS-TypeI Cas3_0_I | AMP-AM, PIP, TIC, PIP-TAZ, AMC, CXM, CIX, CTX, CAZ, FEP, AZT, NOR, CIP, LEV, TOB, AMI, TMP, TMP-SXT |
| LREC-288  FVL123/22 | O25:H4 | 131/43 | 85838 | 40-30 | *bla*_CTX-M-15_, *bla*_OXA-1_, *aac(3)-Iia*, *aac(6’)-Ib-cr*, *mdf(A*), *catB3*, *sitABCD*  ***gyrA* p.S83L, *gyrA* p.D87N, *parC* p.S80I, *parC* p.E84V, *parE* p.I529L** | IncF [F36:A4:B1] | *anr, chuA, cnf1, csgA, fdeC, fimH, fyuA, gad, hlyA, hra, iha, irp2, iss, iucC, iutA, kpsE, kpsMII K5, nlpl, ompT, papA F43, papC, sat, sitA, terC, traJ, traT, usp, yehA, yehB, yehC, yehD, yfcV* | IS30 (*iutA, sat, iha, iucC*), IS629 (*iutA, sat, iha, iucC*), ISKpn37 (*cnf1*, *hlyA*), MITEEc1 (*terC*) | - | AMP-AM, PIP, TIC, AMC, CXM, CIX, CTX, CAZ, FEP, AZT, NOR, CIP, LEV, GEN, TOB |
| **LREC-289**  FVL125/22 | O25:H4 | 131/43 | 71301 | 40-30 | *bla*_CTX-M-15_**,** *bla*_OXA-1_, *aac(3)-Iia,* *aac(6’)-Ib-cr*, *mdf(A)*, *catB3*, *tet(A)*, *sitABCD*  ***gyrA* p.S83L, *gyrA* p.D87N, *parC* p.S80I, *parC* p.E84V, *parE* p.I529L** | IncF [F31:A4:B1] | *anr, chuA, cnf1, csgA, fdeC, fimH, fyuA, gad, hlyA, hra, iha, irp2, iss, iucC, iutA, kpsE, kpsMII K5, nlpl, ompT, papA F43, papC, sat, senB, shiB, sitA, terC, traJ, traT, usp, yehA, yehB, yehC, yehD, yfcV* | ISSfl10 (*iucC, sat, iutA*), ISKpn37 (*cnf1, hlyA*), MITEEc1 (*terC*) | CAS-TypeI Cas3_0_I | AMP-AM, PIP, TIC, AMC, CXM, CIX, CTX, CAZ, FEP, AZT, NOR, CIP, LEV, GEN, TOB |
| LREC-293  FVL37/22 | O25:H4 | 131/43 | 135474 | 40-602 | *bla_CTX-M-15_****,*** *aadA5, mph(A), sul1, tet(A), dfrA17, sitABCD, qacE*  ***gyrA* p.S83L, *gyrA* p.D87N, *parC* p.S80I, *parC* p.E84V, *parE* p.I529L** | IncF [F36:A4:B1]  Col156 | *afaA, afaC, afaD, anr, chuA, csgA, fdeC, fimH, fyuA, gad, hha, hra, iha, irp2, iss, iucC, kpsE, kpsMII_K5, nfaE, nlpI, ompT, papA_F43, sat, senB, shiA, shiB, sitA, terC, traJ, traT,usp, yehA, yehB, yehC, yehD, yfcV* | IS4 (*terC, kpsE, kpsMII_K5*), IS30 (*sat*), IS629 (*iucC, papA_F43*), IS6100 (*dfrA17*, *qacE*, *mph(A)*, *sul1*, *aadA5*), ISEc1 (*gad*), MITEEc1 (*terC*), ISEc53 (*fimH*, *usp*), Col156 (*senB*), MITEEc1 (*ompT, csgA, fdeC*), MITEEc1 (*yfcV*) | - | AMP-AM, PIP, TIC, CXM, CIX, CTX, CAZ, FEP, AZT, NOR, CIP, LEV, TMP, TMP-SXT |

^1^Isolate and genome (LREC) identification. In bold, those genomes also analyzed in García et al., (2023). ^2^O and H antigen prediction with SerotypeFinder 2.0; ^3^Sequence types (ST#1 and ST#2) based on two different MLST schemes were applied: *E. coli* #1 and *E. coli* #2, respectively, and retrieved with MLST 2.0.4. ^4^Core genome ST obtained with cgMLSTFinder1.1. software run against the Enterobase database. ^5^Clonotypes, ^6^acquired antimicrobial resistance genes and/or chromosomal mutations, ^7^replicon/plasmid sequence types, ^8^virulence genes and ^9^the mobile genetic elements associated with AMR and virulence traits were also predicted using: Chtyper 1.0, ResFinder 4.1, PlasmidFinder 2.1, pMLST 2.0, VirulenceFinder 2.0 and MobileElementFinder 1.03 online tools at the Center of Genomic Epidemiology (<http://www.genomicepidemiology.org/services/>), respectively. ^10^CRISPRCasFinder software (<https://crisprcas.i2bc.paris-saclay.fr/>) was used to identify and type CRISPR and Cas systems within the genomes.

^6^Resistome: Acquired resistance genes: beta-lactam: *bla*_TEM-1A_, *bla*_TEM-1B_, *bla*_OXA-1_, *bla*_CTX-M-15_, aminoglycosides: *aac(3)-Iia, aadA2, aadA5, aph(3’’)-Ib, aph(6)-Id*; phenicols: *catB3*; fluoroquinolones: *aac(6’)-Ib-cr*, *qnrS1*; macrolides: *mdf(A), mph(A)*; sulphonamides: *sul1*, *sul2*; tetracycline: *tet(A)*; trimethoprim: *dfrA12, dfrA14, dfrA17*; quaternary ammonium compounds: *qacE* , peroxide: *sitABCD* (mediates transport of iron and manganese and resistance to hydrogen peroxide). Point mutations: quinolones and fluoroquinolones: *gyrA* S83L: TCG-TTG, *gyrA* D87N: GAC-AAC, *parC* E84V: GAA-GTA, *parC* S80I: AGC-ATC, *parE* I529: ATT-CTT.

^7^Plasmid STs: “∗” indicates alleles with less than 100% but >95% identity and 100% coverage.

^8^Virulence determinants: anr: *araC* negative regulator; *afaA*: transcriptional regulator; *afaC*: outer membrane usher protein; *afaD*: afimbrial adhesion; *chuA*: outer membrane hemin receptor; *cnf1*: cytotoxic necrotizing factor; *colE2*-like: colicin lysis protein precursor E2-like; *csgA*: curlin major subunit CsgA; *faeC*: F4(K88) minor fimbrial subunits; *faeD*: F4 (K88) usher; *faeF*: F4 (K88) minor fimbrial subunit; *faeH*: F4 (K88) fimbrial minor subunit; *fael*: F4 (K88) fimbrial minor subunit; *fdeC*: intimin-like adhesin FdeC; *fimH*: type 1 fimbriae; *fyuA*: siderophore receptor; *gad*: glutamate decarboxylase; *hha*: hemolysin expression modulator Hha (previous *rmoA*); *hra*: heat-resistant agglutinin; *iha*: adherence protein; *irp2*: high molecular weight protein 2 non-ribosomal peptide synthetase; *iss*: increased serum survival; *iucC*: aerobactin synthetase; *iutA*: ferric aerobactin receptor; *kpsE*: capsule polysaccharide export inner-membrane protein; *kpsMII_K5:* polysialic acid transport protein group 2 capsule; *nlpl*: lipoprotein Nlpl precursor; *nfaE*: diffuse adherence fibrillar adhesin gene; *ompT*: outer membrane protease (protein protease 7); *papA_F43*: major pilin subunit F43; *papC*: outer membrane usher P fimbriae; *sat*: secreted autotransporter toxin; *senB*: plasmid-encoded enterotoxin; *shiB*: homologs of the Shigella flexneri SHI-2 pathogenicity island gene shiA; *sitA*: iron transport protein; *terC*: tellurium ion resistance protein; *traJ*: protein TraJ (positive regulator of conjugal transfer operon); *traT*: outer membrane protein complement resistance, *usp*: uropathogenic specific protein; *yehA*: outer membrane lipoprotein, YHD fimbriael cluster; *yehB*: usher, YHD fimbriael cluster; *yehC*: chaperone, YHD fimbriael cluster; *yehD*: major pilin subunit YHD fimbriael cluster; *yfcV*: fimbrial protein.

^11^Phenotypic resistances were interpreted according to EUCAST 2022 breakpoints. Antimicrobial abbreviation: ampicillin/amoxicillin: AMP-AM, Piperacillin: PIP, Ticarcillin: TIC, piperacillin/tazobactam: PIP-TAZ, amoxicillin/clavulanic acid: AMC, Cefuroxime: CXM, Cefixime: CIX, Cefotaxime: CTX, Ceftazidime: CAZ, Cefepime: FEP, Ceftolozane/Tazobactam: CTN-TAZ, Aztreonam: AZT, Norfloxacin: NOR, Ciprofloxacin: CIP, Levofloxacin: LEV, Gentamicin: GEN, Tobramycin: TOB, Amikacin: AMI, Trimethoprim: TMP, Trimethoprim-Sulfamethoxazole: TMP-SXT.

**Table S10.** Pairwise distance matrix based on the SNP counts per substitution within the core genome of the 12 ESBL-producing ST131 *E. coli* using LREC-280 genome as a reference

|  | **LREC-**  **278** | **LREC-**  **279** | **LREC-**  **281** | **LREC-**  **283** | **LREC-**  **284** | **LREC-**  **285** | **LREC-**  **286** | **LREC-**  **287** | **LREC-**  **288** | **LREC-**  **289** | **LREC-**  **293** | **LREC-**  **280** |
| --- | --- | --- | --- | --- | --- | --- | --- | --- | --- | --- | --- | --- |
| **LREC-**  **278** | 0 | 384 | 133 | 449 | 428 | 382 | 458 | 449 | 113 | 101 | 94 | 364 |
| **LREC-**  **279** | 384 | 0 | 400 | 237 | 215 | 75 | 337 | 272 | 416 | 406 | 389 | 231 |
| **LREC-**  **281** | 133 | 400 | 0 | 476 | 451 | 401 | 479 | 432 | 152 | 142 | 139 | 384 |
| **LREC-**  **283** | 449 | 237 | 476 | 0 | 105 | 217 | 381 | 340 | 478 | 466 | 462 | 276 |
| **LREC-**  **284** | 428 | 215 | 451 | 105 | 0 | 194 | 344 | 299 | 459 | 445 | 436 | 236 |
| **LREC-**  **285** | 382 | 75 | 401 | 217 | 194 | 0 | 306 | 269 | 407 | 403 | 386 | 214 |
| **LREC-**  **286** | 458 | 337 | 479 | 381 | 344 | 306 | 0 | 375 | 487 | 473 | 464 | 289 |
| **LREC-**  **287** | 449 | 272 | 432 | 340 | 299 | 269 | 375 | 0 | 434 | 444 | 453 | 223 |
| **LREC-**  **288** | 113 | 416 | 152 | 478 | 459 | 407 | 487 | 434 | 0 | 96 | 115 | 352 |
| **LREC-**  **289** | 101 | 406 | 142 | 466 | 445 | 403 | 473 | 444 | 96 | 0 | 99 | 358 |
| **LREC-**  **293** | 94 | 389 | 139 | 462 | 436 | 386 | 464 | 453 | 115 | 99 | 0 | 367 |
| **LREC-**  **280** | 364 | 231 | 384 | 276 | 236 | 214 | 289 | 223 | 352 | 358 | 367 | 0 |

Min: 75, Max: 487

**References**

Blanco, M., Alonso, M. P., Nicolas-Chanoine, M.-H., Dahbi, G., Mora, A., Blanco, J. E., et al. (2009). Molecular epidemiology of Escherichia coli producing extended-spectrum β-lactamases in Lugo (Spain): dissemination of clone O25b:H4-ST131 producing CTX-M-15. *J. Antimicrob. Chemother.* 63, 1135–1141. doi: 10.1093/jac/dkp122.

Borowiak, M., Fischer, J., Hammerl, J. A., Hendriksen, R. S., Szabo, I., and Malorny, B. (2017). Identification of a novel transposon-associated phosphoethanolamine transferase gene, mcr-5, conferring colistin resistance in d-tartrate fermenting Salmonella enterica subsp. enterica serovar Paratyphi B. *J. Antimicrob. Chemother.* 72, 3317–3324. doi: 10.1093/jac/dkx327.

Clermont, O., Bonacorsi, S., and Bingen, E. (2000). Rapid and simple determination of the Escherichia coli phylogenetic group. *Appl. Environ. Microbiol.* 66, 4555–8. Available at: http://www.ncbi.nlm.nih.gov/pubmed/11010916

Clermont, O., Christenson, J. K., Denamur, E., and Gordon, D. M. (2013). The Clermont Escherichia coli phylo-typing method revisited: Improvement of specificity and detection of new phylo-groups. *Environ. Microbiol. Rep.* doi: 10.1111/1758-2229.12019.

Clermont, O., Dixit, O. V. A., Vangchhia, B., Condamine, B., Dion, S., Bridier-Nahmias, A., et al. (2019). Characterization and rapid identification of phylogroup G in Escherichia coli, a lineage with high virulence and antibiotic resistance potential. *Environ. Microbiol.* 21, 3107–3117. doi: 10.1111/1462-2920.14713.

Clermont, O., Lavollay, M., Vimont, S., Deschamps, C., Forestier, C., Branger, C., et al. (2008). The CTX-M-15-producing Escherichia coli diffusing clone belongs to a highly virulent B2 phylogenetic subgroup. *J. Antimicrob. Chemother.* 61, 1024–8. doi: 10.1093/jac/dkn084.

Dahbi, G., Mora, A., Mamani, R., López, C., Alonso, M. P., Marzoa, J., et al. (2014). Molecular epidemiology and virulence of Escherichia coli O16:H5-ST131: comparison with H30 and H30-Rx subclones of O25b:H4-ST131. *Int. J. Med. Microbiol.* 304, 1247–57. doi: 10.1016/j.ijmm.2014.10.002.

García-Meniño, I., García, V., Mora, A., Díaz-Jiménez, D., Flament-Simon, S. C., Alonso, M. P., et al. (2018). Swine enteric colibacillosis in Spain: Pathogenic potential of mcr-1 ST10 and ST131 E. Coli Isolates. *Front. Microbiol.* 9. doi: 10.3389/fmicb.2018.02659.

García, V., Lestón, L., Parga, A., García-Meniño, I., Fernández, J., Otero, A., et al. (2023). Genomics, biofilm formation and infection of bladder epithelial cells in potentially uropathogenic Escherichia coli (UPEC) from animal sources and human urinary tract infections (UTIs) further support food-borne transmission. *One Heal. (Amsterdam, Netherlands)* 16. doi: 10.1016/J.ONEHLT.2023.100558.

Johnson, J. R., Gajewski, A., Lesse, A. J., and Russo, T. A. (2003). Extraintestinal pathogenic Escherichia coli as a cause of invasive nonurinary infections. *J. Clin. Microbiol.* 41, 5798–802. Available at: http://www.ncbi.nlm.nih.gov/pubmed/14662987

Johnson, J. R., and O’Bryan, T. T. (2004). Detection of the Escherichia coli group 2 polysaccharide capsule synthesis Gene kpsM by a rapid and specific PCR-based assay. *J. Clin. Microbiol.* 42, 1773–6. Available at: http://www.ncbi.nlm.nih.gov/pubmed/15071046

Johnson, J. R., Russo, T. A., Tarr, P. I., Carlino, U., Bilge, S. S., Vary, J. C., et al. (2000). Molecular epidemiological and phylogenetic associations of two novel putative virulence genes, iha and iroN(E. coli), among Escherichia coli isolates from patients with urosepsis. *Infect. Immun.* 68, 3040–7. Available at: http://www.ncbi.nlm.nih.gov/pubmed/10769012

Johnson, J. R., and Stell, A. L. (2000). Extended Virulence Genotypes of *Escherichia coli* Strains from Patients with Urosepsis in Relation to Phylogeny and Host Compromise. *J. Infect. Dis.* 181, 261–272. doi: 10.1086/315217.

Le Bouguenec, C., Archambaud, M., and Labigne, A. (1992). Rapid and specific detection of the pap, afa, and sfa adhesin-encoding operons in uropathogenic Escherichia coli strains by polymerase chain reaction. *J. Clin. Microbiol.* 30, 1189–93. Available at: http://www.ncbi.nlm.nih.gov/pubmed/1349900

Lescat, M., Clermont, O., Woerther, P. L., Glodt, J., Dion, S., Skurnik, D., et al. (2013). Commensal Escherichia coli strains in Guiana reveal a high genetic diversity with host-dependant population structure. *Environ. Microbiol. Rep.* 5, 49–57. doi: 10.1111/j.1758-2229.2012.00374.x.

Mora, A., Herrrera, A., López, C., Dahbi, G., Mamani, R., Pita, J. M., et al. (2011). Characteristics of the Shiga-toxin-producing enteroaggregative Escherichia coli O104:H4 German outbreak strain and of STEC strains isolated in Spain. *Int. Microbiol.* 14, 121–41. doi: 10.2436/20.1501.01.142.

Mora, A., Viso, S., López, C., Alonso, M. P., García-Garrote, F., Dabhi, G., et al. (2013). Poultry as reservoir for extraintestinal pathogenic Escherichia coli O45: K1: H7-B2-ST95 in humans. *Vet. Microbiol.* 167, 506–512. doi: 10.1016/j.vetmic.2013.08.007.

Moulin-Schouleur, M., Schouler, C., Tailliez, P., Kao, M.-R., Brée, A., Germon, P., et al. (2006). Common virulence factors and genetic relationships between O18:K1:H7 Escherichia coli isolates of human and avian origin. *J. Clin. Microbiol.* 44, 3484–92. doi: 10.1128/JCM.00548-06.

Pérez-Pérez, F. J., and Hanson, N. D. (2002). Detection of plasmid-mediated AmpC beta-lactamase genes in clinical isolates by using multiplex PCR. *J. Clin. Microbiol.* 40, 2153–62. Available at: http://www.ncbi.nlm.nih.gov/pubmed/12037080

Rebelo, A. R., Bortolaia, V., Kjeldgaard, J. S., Pedersen, S. K., Leekitcharoenphon, P., Hansen, I. M., et al. (2018). Multiplex PCR for detection of plasmid-mediated colistin resistance determinants, mcr-1, mcr-2, mcr-3, mcr-4 and mcr-5 for surveillance purposes. *Euro Surveill.* 23. doi: 10.2807/1560-7917.ES.2018.23.6.17-00672.

Saladin, M., Cao, V. T. B., Lambert, T., Donay, J.-L., Herrmann, J.-L., Ould-Hocine, Z., et al. (2002). Diversity of CTX-M beta-lactamases and their promoter regions from Enterobacteriaceae isolated in three Parisian hospitals. *FEMS Microbiol. Lett.* 209, 161–8. doi: 10.1111/j.1574-6968.2002.tb11126.x.

Simarro, E., Navarro, F., Ruiz, J., Miró, E., Gómez, J., and Mirelis, B. (2000). Salmonella enterica serovar virchow with CTX-M-like beta-lactamase in Spain. *J. Clin. Microbiol.* 38, 4676–8. Available at: http://www.ncbi.nlm.nih.gov/pubmed/11101623

Spurbeck, R. R., Dinh, P. C., Walk, S. T., Stapleton, A. E., Hooton, T. M., Nolan, L. K., et al. (2012). Escherichia coli isolates that carry vat, fyuA, chuA, and yfcV efficiently colonize the urinary tract. *Infect. Immun.* 80, 4115–22. doi: 10.1128/IAI.00752-12.

Tóth, I., Hérault, F., Beutin, L., and Oswald, E. (2003). Production of cytolethal distending toxins by pathogenic Escherichia coli strains isolated from human and animal sources: establishment of the existence of a new cdt variant (Type IV). *J. Clin. Microbiol.* 41, 4285–91. Available at: http://www.ncbi.nlm.nih.gov/pubmed/12958258

Weissman, S. J., Johnson, J. R., Tchesnokova, V., Billig, M., Dykhuizen, D., Riddell, K., et al. (2012). High-Resolution Two-Locus Clonal Typing of Extraintestinal Pathogenic Escherichia coli. doi: 10.1128/AEM.06663-11.

Wirth, T., Falush, D., Lan, R., Colles, F., Mensa, P., Wieler, L. H., et al. (2006). Sex and virulence in Escherichia coli : an evolutionary perspective. *Mol. Microbiol.* 60, 1136–1151. doi: 10.1111/j.1365-2958.2006.05172.x.

Yamamoto, S., Terai, A., Yuri, K., Kurazono, H., Takeda, Y., and Yoshida, O. (1995). Detection of urovirulence factors in Escherichia coli by multiplex polymerase chain reaction. *FEMS Immunol. Med. Microbiol.* 12, 85–90. doi: 10.1111/j.1574-695X.1995.tb00179.x.

**
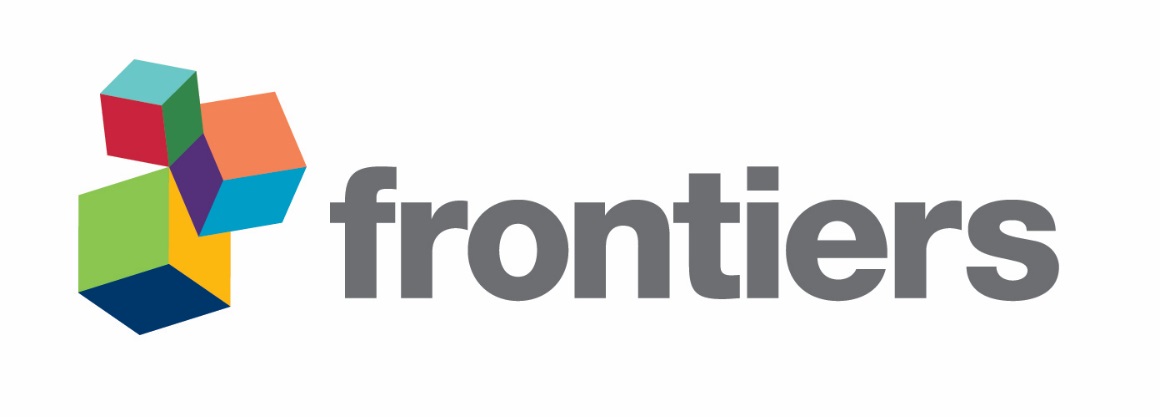
**
